# SiBot: A hybrid framework for fine-grained semantic attribution of AI-generated text and its production deployment

**DOI:** 10.1371/journal.pone.0357405

**Published:** 2026-09-08

**Authors:** Md. Sadiq Iqbal, Mohammod Abul Kashem

**Affiliations:** Department of Computer Science and Engineering, Dhaka University of Engineering and Technology, Gazipur, Bangladesh; The University of Lahore, PAKISTAN

## Abstract

Large language models such as ChatGPT, Gemini, Claude and DeepSeek now generate text that is routinely indistinguishable from human writing, yet virtually every deployed detector answers only one binary question: human or AI. This is the wrong question for the settings that matter most. Digital forensics needs to know *which* model produced a text; academic-integrity adjudication needs to know whether a human was involved at all; and neither can be answered by a single probability, least of all for the hybrid human-AI drafts that now dominate real-world writing. This paper addresses that gap with SiBot, an end-to-end framework that reformulates AI-text detection as fine-grained multi-class source attribution and carries it through to public deployment. We construct two purpose-built corpora using a controlled bank of 3,000 prompts spanning six subject domains and five task types: a 66,000-sample corpus covering 22 AI sources, and a 30,000-sample corpus covering 10 classes that explicitly models five pure sources and four human-AI collaboration workflows; both are publicly released as the TXD-22 benchmark dataset. On the 22-class corpus we benchmark twelve feature-classifier combinations and find that TF-IDF with Random Forest attains 68.2% accuracy and an AUC of 0.742. On the 10-class corpus we benchmark fifteen classical, deep-learning and fine-tuned transformer baselines, and propose a hybrid architecture that fuses sparse TF-IDF lexical features with DeBERTa contextual embeddings at the feature level before Random Forest classification. The proposed hybrid attains 92.38% accuracy, 92.41% precision, 92.38% recall and 92.31% F1 under prompt-aware 10-fold cross-validation, a 17.73-point gain over the strongest fine-tuned transformer (DeBERTa, 74.65%) and a 34.30-point gain over TF-IDF with Random Forest alone, with non-overlapping 95% confidence intervals. An ablation confirms that both branches are necessary, and SHAP and LIME analyses expose the lexical evidence behind individual decisions. Because the 10-class hybrid is both substantially more accurate and interpretable, it is the model served by the live SiBot platform; the 22-class model is reported here as a research benchmark, and extending the deployment to all 22 classes is left to future work. In a controlled three-way benchmark against QuillBot, Turnitin, ZeroGPT and TextGuard, SiBot is the only system that names the generating model of AI text (Gemini, 96.7% confidence) and the only one that recognises hybrid provenance (97.7% confidence, against verdicts ranging from 0% to 76% among the commercial tools on the identical input).

## 1. Introduction

The rapid democratization of large language models (LLMs) such as ChatGPT, Gemini, Claude, and Llama has revolutionized content creation across domains, yet it has simultaneously introduced pressing challenges related to academic integrity, misinformation, and digital trust [1,2]. While current detection tools attempt to address these concerns, they are predominantly limited to binary human-versus-AI classification, an approach that fails to capture the nuanced stylistic fingerprints of individual models, cannot distinguish between different AI sources for forensic purposes, and struggles with the increasingly prevalent reality of hybrid human-AI content [3,4]. Moreover, a substantial gap persists between experimental detection models developed in academic settings and their deployment as practical, scalable tools accessible to educators, journalists, and the public [5].

The specific problem this work addresses can be stated precisely. Given an arbitrary passage of text, existing systems return a scalar AI-likelihood. What forensic, editorial and educational users actually require is a distribution over provenance classes: which model, if any, produced the text, and to what extent a human participated in its production. Three obstacles have prevented this reformulation from being realized in practice. First, no public corpus has simultaneously covered a large number of contemporary generators *and* explicitly enumerated multi-stage human-AI collaboration workflows. Second, the two dominant feature families have been treated as competitors rather than complements: sparse lexical statistics capture the surface idiosyncrasies that distinguish generators but ignore context, whereas transformer embeddings capture context but collapse the surface signal that carries the attribution evidence. Third, the few systems that do attempt attribution remain research prototypes and are never exposed to real users under real latency and cost constraints.

This paper presents SiBot, a complete research-to-production system designed to address these limitations through several integrated contributions. We introduce a comprehensive dataset comprising 66,000 text samples spanning 22 distinct classes, including 21 individual AI sources ranging from closed-source models like GPT-4 to open-source alternatives like Llama, plus a hybrid human-AI category, representing one of the most extensive multi-class corpora for AI source attribution. Through rigorous evaluation of feature extraction methods (TF-IDF, ALBERT, RoBERTa) paired with various classifiers, we demonstrate that the combination of TF-IDF vectorization with a Random Forest classifier achieves optimal performance at 68.2% accuracy across all 22 categories [6,7]. We then show that this ceiling is a property of the feature representation rather than of the task, and lift it substantially with a second corpus and a second architecture. A 30,000-sample, 10-class corpus is constructed in which the four principal human-AI collaboration workflows are represented as first-class categories, and a hybrid model is proposed that concatenates TF-IDF and DeBERTa representations before Random Forest classification. This hybrid attains 92.38% accuracy, a 24.18-point absolute improvement over the 22-class configuration and a 17.73-point improvement over the strongest fine-tuned transformer evaluated on the same data. Because this configuration is the one that meets the accuracy and interpretability requirements of a public service, it is the model integrated into the deployed SiBot platform. The 22-class model remains an important research benchmark and is reported in full, but scaling the deployment to all 22 classes is deliberately deferred to future work, for the reasons set out in Section 4.9.

The deployed model forms the core of SiBot’s three-tier production architecture: a serialized classifier served by a Fast API backend containerized with Docker and deployed on Hugging Face Spaces, complemented by a React TypeScript frontend utilizing Tailwind CSS and shadcn/ui, with Supabase providing authentication, PostgreSQL database persistence, and comprehensive user management through Row Level Security [8–10]. Beyond its technical architecture, SiBot introduces advanced functionality absent from existing detection tools, including exact source attribution that identifies specific AI models rather than merely flagging AI-generated content [11–13], sentence-by-sentence analysis capable of decomposing mixed-origin documents, PDF upload and text extraction capabilities, batch prediction for multiple inputs, and shareable report generation accessible without user registration. The system additionally features a complete administrator panel for managing users, roles, rate limits, and audit logging.

### 1.1. Novelty and contributions

The novelty of this work is not the use of TF-IDF, of DeBERTa, or of Random Forest individually, all of which are established. It lies in three specific claims, each of which is tested empirically in Section 4. First, that lexical and contextual evidence for source attribution are complementary rather than redundant, and that fusing them at the feature level rather than at the decision level yields a gain far larger than either branch contributes alone: 92.38% against 58.08% for TF-IDF with Random Forest and 74.65% for a fine-tuned DeBERTa on identical data and splits. Second, that human-AI collaboration is separable into distinguishable workflow classes rather than being a single undifferentiated ‘hybrid’ bucket; the proposed model separates AI-generated-then-human-edited, AI-generated-then-AI-refined, human-written-then-AI-refined, and the three-stage combination, at per-class recalls between 91.8% and 92.4%. Third, that attribution-grade granularity is achievable within production latency and cost constraints, which we demonstrate by serving the model publicly rather than reporting offline numbers only. The specific contributions are:

- This study establishes a comprehensive framework for multi-class AI source attribution, moving beyond binary detection to enable precise identification of specific language models.
- A hybrid feature-fusion architecture (TF-IDF + DeBERTa + Random Forest) is proposed and shown, through a component-wise ablation, to derive its performance from the interaction of its branches rather than from either branch alone.
- The introduction of a 66,000-sample dataset spanning 22 distinct AI sources plus hybrid content provides an unprecedented resource for advancing forensic analysis of synthetic text, complemented by a 30,000-sample, 10-class corpus in which four human-AI collaboration workflows are explicitly modelled as separate classes. Both corpora are publicly released as the TXD-22 benchmark dataset under DOI 10.17632/prcjcggtjf.1 [14], so that the results reported here are directly reproducible and the corpus is available for use by other groups.
- A documented prompt-engineering protocol, a prompt-aware splitting strategy that prevents prompt leakage between training and test partitions, and representative dataset examples are provided so that the corpus construction is reproducible.
- Post-hoc explainability through SHAP and LIME is integrated into the evaluation, making the lexical basis of each attribution decision inspectable rather than opaque.
- A quantitative benchmark against four commercial detectors (QuillBot, Turnitin, ZeroGPT, TextGuard) across AI-generated, human-written and hybrid inputs demonstrates that no existing tool provides source attribution or hybrid recognition at all.
- The development of a fully functional three-tier web application bridges the critical gap between experimental detection models and practical, scalable tools accessible to educators, journalists, and the public.

This paper is organized as follows. Section 2 reviews related work in AI text detection, stylometry, and production ML systems, including recent 2024–2026 contributions. Section 3 details the methodology, covering dataset creation, feature extraction, the proposed hybrid architecture, the validation protocol, and the complete system architecture of SiBot. Section 4 presents experimental results, ablation, statistical validation, per-class analysis, efficiency measurements and system performance. Section 5 discusses the implications of our findings, the quantitative comparison against commercial detectors, and the ethical considerations raised by attribution technology. Section 6 concludes the paper and outlines directions for future work.

## 2. Literature review

The challenge of distinguishing human-written text from AI-generated content has evolved rapidly alongside advances in large language models. Early detection methods relied heavily on statistical and linguistic features, while contemporary approaches leverage deep learning architectures and ensemble methods. This section reviews the existing body of work, highlighting the progression from binary detection to fine-grained attribution, the role of stylometry, and the emergence of production-ready systems. Subsections 2.1 to 2.4 trace this progression chronologically; Section 2.5 surveys the most recent 2024–2026 literature; and Section 2.6 states explicitly how the present contribution differs from each of these strands.

### 2.1. Binary AI text detection

The majority of existing research focuses on binary classification, attempting to answer whether a given text was written by a human or an AI. A seminal work by OpenAI [15] introduced a classifier trained to distinguish GPT-2 generated text from human-written text, achieving notable success on in-domain data but demonstrating significant limitations in generalization. Subsequent studies expanded on this foundation. Gehrmann et al. [16] developed GLTR (Giant Language Model Test Room), a tool that uses the statistical properties of language models to highlight passages that are likely machine-generated by visualizing the ranking of each word under a model’s predictive distribution. This approach provided interpretable results but was limited to the specific model it was calibrated against. Mitchell et al. [17] proposed DetectGPT, a zero-shot method for detecting machine-generated text that exploits the observation that generated text tends to occupy negative curvature regions of the model’s log-probability function. This method achieved high performance without requiring a separate training dataset but was computationally expensive. More recently, OpenAI discontinued its AI classifier due to its low accuracy, highlighting the inherent difficulty of the task [18]. Several commercial tools have emerged, including ZeroGPT [19] and QuillBot’s AI detector [20], which provide user-friendly interfaces but maintain proprietary, often non-transparent, detection methodologies. A common limitation across these binary tools is their inability to provide granular insights or to differentiate between outputs from distinct AI models.

### 2.2. Stylometry and linguistic fingerprints

The field of stylometry, which analyzes linguistic style to attribute authorship, provides a theoretical foundation for fine-grained AI source attribution [21]. Early work by Holmes [22] established that authors have unique, quantifiable stylistic features such as function word frequency, sentence length distribution, and vocabulary richness. These principles have been successfully applied to distinguish between human authors and have more recently been extended to AI models. Research by Liang et al. [23] investigated the concept of “neural fingerprints” in large language models, demonstrating that different models exhibit distinct stylistic patterns that can be captured through simple features like token distributions and n-gram frequencies. Similarly, Krishna et al. [24] explored paraphrasing strategies and showed that even when content is rewritten, certain stylistic signatures of the originating model can persist. Seraj et al. [25] applied stylometric analysis to distinguish between human and ChatGPT essays, achieving high accuracy using traditional linguistic features. Kumarage and Liu [26] made this explicit for the attribution setting, extracting sixty lexical, syntactic and structural stylometric features and showing that they carry enough signal to trace text back to its originating LLM across both proprietary and open-source model families. These studies collectively suggest that while the semantic content of different models may converge, their stylistic fingerprints remain distinct, providing a basis for multi-class classification.

### 2.3. Multi-class attribution and model fingerprinting

The move from binary detection to multi-class source attribution is a more recent but crucial development. Recent work has begun to explore the possibility of not just detecting AI-generated text, but identifying which specific AI model generated it. This is particularly important for forensic analysis and understanding model biases. Uchendu et al. [27] introduced the concept of “model attribution” and presented a methodology using n-gram features and supervised classifiers to identify the source model of a text, achieving strong results on a corpus of texts from a limited set of models. Fröhling and Zubiaga [28] extended this by focusing on the linguistic differences between GPT-2, GPT-3, and human-written text, demonstrating that transformer-based models like BERT could be used for attribution. Pagnoni et al. [29] introduced the “Ivy” dataset and explored fine-grained detection of text from multiple LLMs, finding that even models from the same family could be differentiated with reasonable accuracy. Ghosal et al. [30] took a different approach by focusing on “semantic drift,” analyzing how the content and style of generated text vary across different models when given identical prompts. Their findings align with our own, suggesting that stylistic features are more discriminative than purely semantic ones. These studies provide the critical foundation for SiBot, yet none have combined this research with a comprehensive, production-ready deployment.

### 2.4. Production ML systems for NLP

A significant gap in the literature exists between high-accuracy research models and their deployment as robust, scalable applications. This gap is often referred to as the “last mile” problem in machine learning engineering [31]. Several works have outlined best practices for deploying ML models, including model serialization, containerization with Docker, and the use of cloud platforms [32]. Huyen [33] provides a comprehensive framework for designing machine learning systems that are reliable, scalable, and maintainable, emphasizing the importance of monitoring, versioning, and infrastructure. Specific to NLP, frameworks like Hugging Face Transformers have democratized access to pre-trained models, while platforms like Hugging Face Spaces simplify deployment [34]. FastAPI has emerged as a popular choice for serving ML models due to its high performance and automatic OpenAPI documentation [35]. The use of Supabase (PostgreSQL) with Row Level Security for user management and authentication is a modern approach to building secure, data-driven applications without custom backend code [36]. SiBot leverages these modern engineering practices to deliver its research contributions as a public, accessible tool.

### 2.5. Recent advances (2024–2026)

The field has moved substantially since the foundational work reviewed above, and three developments in particular bear directly on the design decisions taken in this paper. The first is the maturation of *robustness benchmarking*. Dugan et al. [37] released RAID, comprising more than six million generations across eleven models, eight domains, eleven adversarial attacks and four decoding strategies, and demonstrated that both open-source and commercial detectors degrade sharply under adversarial paraphrasing, altered sampling strategies and unseen generators. He et al. [38] reached a compatible conclusion with MGTBench. The practical implication for the present work is that a detector evaluated only on in-domain data is evaluated optimistically, which motivates the prompt-aware splitting protocol described in Section 3.1.7 and the explicit generalisation analysis in Section 4.7.

The second development is the emergence of *efficient zero-shot detection*. Bao et al. [39] introduced Fast-DetectGPT, replacing the expensive perturbation step of DetectGPT with a conditional probability curvature statistic and achieving roughly two orders of magnitude speed-up, while Hans et al. [40] proposed Binoculars, which contrasts the perplexity of two closely related language models and detects over 90% of ChatGPT generations at a false-positive rate of 0.01% without any training data. These methods are strong binary detectors, but they are structurally unable to perform source attribution: the score they compute is a scalar, and it does not identify a generator. They therefore establish the state of the art for the question this paper argues is the wrong one, and reinforce the case for a multi-class formulation.

The third and most directly relevant development is the consolidation of *attribution as a distinct task*. Huang, Chen and Shu [41] surveyed authorship attribution in the LLM era and formalised precisely the four-way taxonomy this paper operationalises: attributing text to human authors, detecting LLM-generated text, identifying the specific LLM responsible, and classifying text as human-authored, machine-generated, or co-authored by both. La Cava and Tagarelli [42] released OpenTuringBench, an open-model benchmark explicitly targeting both detection and attribution, and Bisztray et al. [43] demonstrated the analogous problem for source code, showing that model-specific stylometric signatures survive in generated C programs. Work on mixed provenance has advanced in parallel: Chen et al. [44] framed the LLM-as-a-coauthor problem and showed that mixed human-machine text is markedly harder to detect than either pure category, and Thai et al. [45] introduced EditLens to quantify the *extent* of AI editing rather than merely its presence. Georgiou [46] showed that automatically extracted linguistic features remain competitive discriminators. Collectively, this recent literature validates the multi-class, hybrid-aware formulation adopted here while leaving the specific combination proposed in this paper (feature-level fusion of sparse lexical and dense contextual representations, evaluated across both a 22-source and a 10-class workflow-aware corpus and served in production) unaddressed.

### 2.6. Gaps in existing literature and SiBot’s position

Despite significant advancements in AI text detection and attribution, several critical gaps remain. First, most research focuses on a small number of AI models (often 2–5), limiting the practical applicability of findings. Second, there is a notable absence of datasets that include hybrid human-AI content, which is increasingly prevalent in real-world scenarios [47]. Third, the evaluation of detection methods is often done on in-domain data, with little attention to generalization across topics or domains. Fourth, and most importantly, there is a profound disconnect between academic models and production systems; researchers rarely deploy their work as functional, user-friendly tools [48]. A fifth gap, evident from the 2024–2026 literature reviewed above, is methodological: statistical and contextual representations are almost always evaluated in competition with one another rather than in combination, so the question of whether their errors are correlated has gone largely untested. SiBot directly addresses these gaps by introducing a dataset of 22 diverse text sources, including a hybrid class, and by deploying the best-performing model as a fully functional three-tier application, thereby demonstrating a complete research-to-production pipeline. Concretely, the present work differs from the closest prior studies in four respects: it evaluates 22 generators where Uchendu et al. [27] and Kumarage and Liu [26] evaluate between two and six; it treats four human-AI collaboration workflows as separate classes where Chen et al. [44] treat mixed authorship as a single category; it fuses lexical and contextual features rather than selecting between them, as all of [27,28,39] and [40] do; and it reports a live, publicly accessible deployment, which none of the cited attribution studies provide.

## 3. Methodology: Research and system design

Our methodology is structured into two interconnected phases: the core AI model development and the production system architecture. Phase 1, described in Section 3.1, covers corpus construction, feature engineering, the proposed hybrid architecture, the validation protocol and model selection. Phase 2, described in Section 3.2, covers the three-tier system that serves the selected model to the public.

### 3.1. Phase 1: AI model development

**Fig 1:**
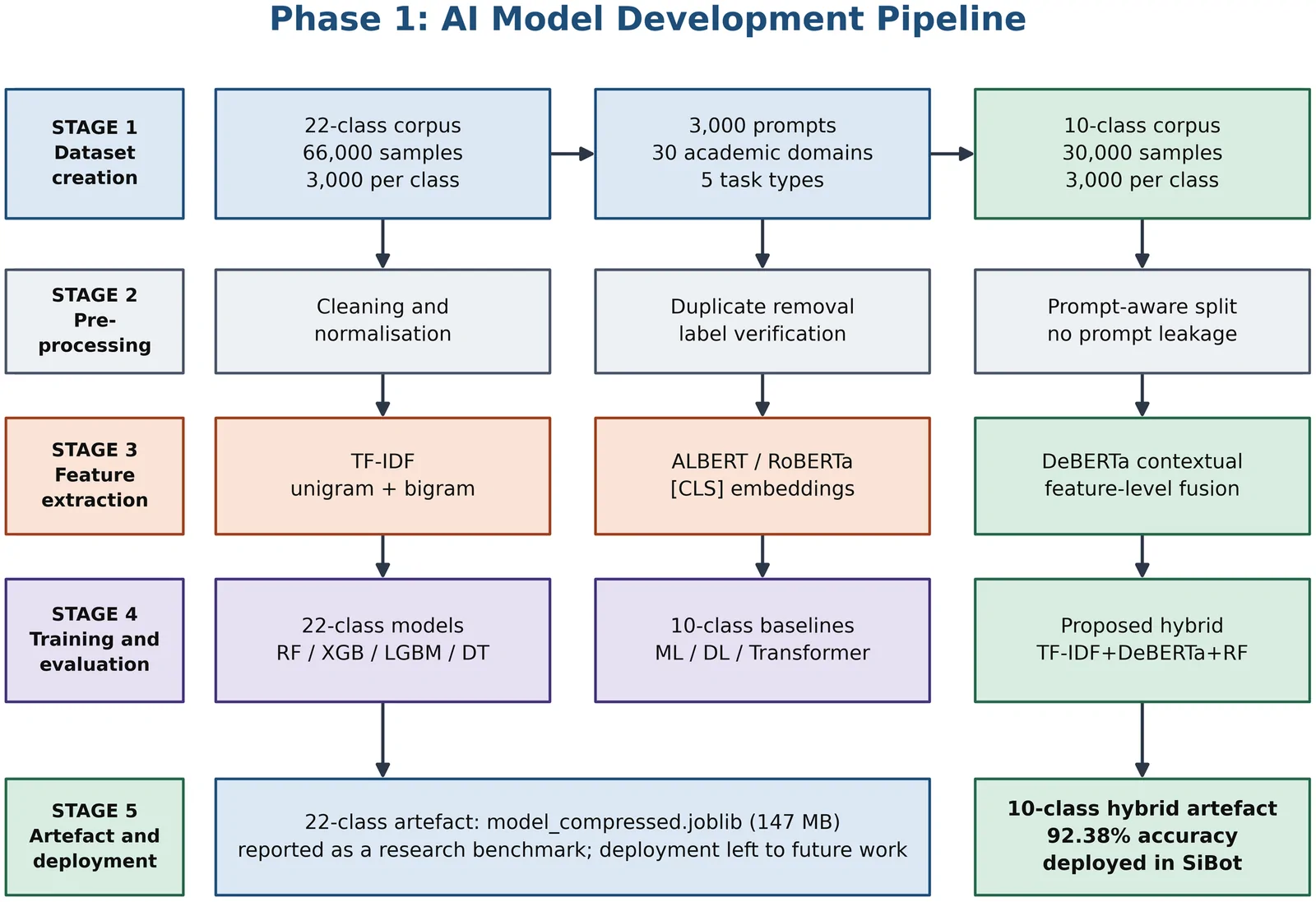
Phase 1 Workflow: AI Model Development Pipeline.

This phase encompasses the end-to-end construction of the classification pipeline, from data collection to the serialization of the final production model. The workflow of phase 1, illustrated in Fig 1, consists of five sequential stages: dataset creation, preprocessing, feature extraction, classifier training and evaluation, and model artifact serialization [49]. Fig 1 has been redrawn at 600 dpi with enlarged typography in response to reviewer comments on figure legibility, and now also shows the second, 10-class experimental track and the fusion branch that were absent from the original pipeline.

#### 3.1.1. Dataset construction and prompt engineering.

The foundation of this study is a novel, purpose-built dataset. We identified 22 distinct AI sources, encompassing a wide range of prominent LLMs and AI assistants including ChatGPT, Grok, Pi, BLACKBOXAI, Nova, Poe, Mistral AI, Perplexity, Z.ai, Gemma, Llama, Claude, Qwen, DeepAI, DeepSeek, Gemini, and Copilot, with a full list maintained in the production system’s/classes endpoint. In addition to the AI sources, we defined a class to capture the complexity of real-world text: “AI-generated and Human-written,” acknowledging that text creation is often an iterative process involving both humans and machines [50]. For each of the 22 classes, we collected 3,000 text samples by posing a single fixed set of 3,000 unique questions or prompts to each AI source in turn, so that every class answers exactly the same questions. The total dataset size is 66,000 samples, ensuring a balanced representation for each class. A second corpus of 30,000 samples across 10 classes was constructed using the same prompt bank and the same protocol, and is used for the hybrid experiments reported in Sections 4.2 to 4.7.

**Definition of diversity.** The term “diverse”, used loosely in the original manuscript, is defined here along three explicit axes. *Topical diversity* means that the prompt bank spans thirty academic domains, each broken down into eight to ten sub-domains, covering engineering, the natural sciences, the social sciences, law, medicine, the humanities and the creative and media disciplines. The full distribution is given in Table 1. This breadth is deliberate and it is central to the validity of the attribution claim: because the identical prompt set is put to every source, every domain is represented in every class in equal proportion, so topic carries no class information and the classifier cannot reach its accuracy by learning subject matter instead of style. *Task diversity* means that within each domain the prompts span five task types that elicit structurally different responses: factual explanation, comparative analysis, procedural or how-to description, opinion or argumentative writing, and summarisation or condensation. Representative examples of each, drawn verbatim from the bank, are: “What is the half-life of a drug and why is it clinically important?” (factual explanation); “How does sociology differ from other social sciences?” (comparative analysis); “Describe the process of market segmentation and how firms apply it.” (procedural description); “How does climate change affect rural communities, and what should be prioritised in response?” (opinion or argumentative); and “Summarise the central claim of utilitarianism as an ethical theory.” (summarisation). *Structural diversity* means that prompt length and specificity were varied deliberately, from single-clause questions of under ten words to multi-constraint instructions exceeding fifty words, so that the corpus is not dominated by a single interrogative register. The complete bank comprises 3,000 unique prompts, and the same 3,000 prompts were put verbatim to every generative source and used to elicit every human-written and hybrid sample, so that the corpora differ across classes only in what produced the response, never in what was asked.

**Table 1:** Distribution of prompts across the thirty academic domains and their sub-domains used in corpus construction. The same 3,000 prompts were put verbatim to every source.

No.	Domain	Sub-domains
1	Computer Science and Engineering	Data structures and algorithms; operating systems; computer networks; DBMS; object-oriented programming; programming languages; software engineering; computer organisation and architecture
2	Electrical and Electronic Engineering	Electrical circuits and networks; electrical machines; power systems; control systems; measurements and instrumentation; power electronics; analog and digital electronics; electromagnetic fields; renewable energy systems
3	Economy	Microeconomics; market structures; macroeconomics; public finance; international economics; development economics; national economy; banking and finance
4	Sociology	Introduction to sociology; founding thinkers and theories; social institutions; social stratification; social change and development; rural and urban sociology; gender and society; social problems; social movements; contemporary issues in Bangladeshi society
5	Business Administration	Principles of management; human resource management; marketing management; financial management; accounting; organisational behaviour; operations management; strategic management; entrepreneurship and innovation; business environment and ethics
6	Pharmacy	Pharmaceutics; pharmaceutical chemistry; pharmacology; pharmacognosy; hospital and clinical pharmacy; pharmaceutical microbiology; biochemistry; pharmaceutical jurisprudence and ethics; industrial pharmacy; public health pharmacy
7	English	Grammar and usage; vocabulary; sentence structure; reading comprehension; writing skills; spoken English; literature; applied English; examination preparation; general knowledge in English
8	Law	Constitutional law; law of contract; criminal law; civil procedure code; criminal procedure code; law of evidence; Muslim and Hindu law; property law; company law; international and miscellaneous law
9	Botany	Plant anatomy and morphology; plant physiology; plant taxonomy and systematics; genetics and plant breeding; ecology and environment; plant pathology; economic botany; plant biotechnology; reproduction in plants
10	Criminology	Introduction to criminology; theories of crime; types of crime; crime and society; criminal justice system; crime prevention and control; research methods; criminology and law; emerging issues
11	Disaster Science	Introduction to disaster science; types of disasters; climate change and global warming; disaster risk reduction; climate resilience and adaptation; risk and vulnerability assessment; policy and governance; preparedness and response; post-disaster recovery
12	Geography	Physical geography; landforms and processes; climatology and meteorology; oceanography and hydrology; human geography; environmental geography; natural hazards; regional geography; geographic techniques and tools
13	Graphic Design	Basics of graphic design; design principles; typography; colour in graphic design; software and tools; branding and identity; layout and composition; digital media and UX/UI design; print design
14	History	The ancient world; the medieval period; the early modern period; the modern period; regional history; movements and ideologies; historical figures; events and revolutions; historiography; contemporary history and globalisation
15	Information Science and Library Management	Information and library science fundamentals; information organisation and retrieval; digital libraries and technologies; information management; library management; information sources and services; research and documentation; emerging trends; professional and ethical issues
16	International Relations	Theories of international relations; actors in IR; diplomacy and foreign policy; international security; international law and organisations; global issues and regional studies; conflict resolution; economic aspects of IR; contemporary issues
17	Journalism	Basics of mass communication; communication theories; journalism basics; media ethics and laws; print media; broadcast media; digital and new media; media management and advertising; international mass communication
18	Nuclear Science and Engineering	Basics of nuclear engineering; nuclear reactions and fission; reactor types; reactor physics; nuclear fuel cycle; heat transfer and thermodynamics; nuclear materials; radiation protection and safety; applications and future trends
19	Philosophy	General philosophy and metaphysics; epistemology; ethics; political philosophy; philosophy of mind; logic and reasoning; philosophy of religion; aesthetics; contemporary philosophy
20	Physics	Mechanics; kinematics; gravitation; thermodynamics; waves and oscillations; optics; electricity and magnetism; modern physics; fluid mechanics; advanced topics
21	Political Science	Political theory and philosophy; political systems and institutions; comparative politics; public administration and policy; political economy; democracy and development; political thought; political processes and behaviour; contemporary issues
22	Psychology	Introduction to psychology; biological bases of behaviour; sensation and perception; learning and conditioning; memory; cognition and intelligence; motivation and emotion; developmental psychology; personality psychology; psychological disorders and therapy
23	Robotics	Robotics fundamentals; sensors and actuators; control systems; kinematics and dynamics; robot programming and languages; robotic perception; mechatronic system design; robotics applications; safety and ethics
24	Statistics	Basics of statistics; probability; probability distributions; sampling and sampling distributions; estimation; hypothesis testing; correlation and regression; analysis of variance; non-parametric tests; advanced applications
25	Television, Film and Photography	Film and media; production process; camera and cinematography; lighting techniques; sound and audio; editing; screenwriting and storytelling; photography basics; advanced photography; industry and careers
26	Theatre and Performance Studies	Theatre history and development; forms and genres; performance theory and criticism; acting techniques; playwriting and script analysis; directing and production; theatre spaces; cultural and political theatre; audience studies
27	Tourism and Hospitality Management	Fundamentals of tourism and hospitality; industry and organisations; tourism marketing; hospitality operations; planning and development; travel and transport; tourism law; human resource management; customer service and experience
28	Women and Gender Studies	Foundations and theories; history of women’s movements; gender and society; sexuality and identity; women’s rights and human rights; gender and work; masculinity studies; representation and media; gender and culture
29	Agriculture	Basic agriculture and fundamentals; crops, pollination and farming types; soil and erosion; animal husbandry and crop management; horticulture and protected cultivation; advanced farming and bio-inputs; seeds and special crops; sustainability and soil testing; pest management; extension and modern techniques
30	Zoology	Principles and classification; animal anatomy and physiology; genetics and evolution; ecology and behaviour; reproduction and development; invertebrate zoology; vertebrate zoology; animal physiology and biochemistry; conservation and applied zoology; research methods

**Prompt engineering protocol.** Prompts were authored and reviewed in four steps. A seed set was drafted per domain and task type; each seed was checked against the others for near-duplication using cosine similarity over TF-IDF vectors, with a 0.85 threshold for rejection; the surviving prompts were then reviewed manually for ambiguity and for wording that might bias a particular model family, such as vendor names or product-specific terminology, which were removed; and finally the same frozen prompt was issued verbatim to every source. Issuing an identical prompt to every source is essential to the validity of the attribution claim: it ensures that measured differences between classes are attributable to generator behaviour rather than to input variation. All generations used each provider’s default decoding settings and no system prompt, so that the captured fingerprint reflects the model as an ordinary user would encounter it. Table 1 gives verbatim examples of the prompts used at each level of the design.

**Class structure and hybrid content generation.** The 10-class corpus comprises five pure AI sources (ChatGPT, Copilot, Gemini, Qwen, Claude), one purely human-written class, and four hybrid classes constructed by a structured multi-stage protocol: AI-generated text subsequently edited or extended by a human; AI-generated text subsequently refined by a second AI pass; human-written text subsequently polished by an AI system; and a three-stage class combining AI generation, AI refinement and final human modification. This design mirrors how documents are actually produced, which is rarely in a single step. Human-written responses were collected from manually curated sources including academic-style writing, student responses and manually authored answers to the same prompts, and every human sample was verified to have been produced without AI assistance.

**Quality control and bias mitigation.** A standardised pipeline removed duplicate and near-duplicate entries using similarity thresholding, discarded incomplete or truncated generations and refusal responses, and normalised spacing, punctuation and character encoding. Every sample was re-checked for correct class labelling. Two specific sources of prompt-induced bias were addressed. Because the same prompts were issued to every source, any topical signal is shared across classes and therefore cannot by itself drive attribution; and because prompts were screened to remove vendor-specific phrasing, no class receives a lexical cue from the prompt itself. Class balance was enforced by construction at 3,000 samples per class in both corpora, which removes the majority-class bias that would otherwise complicate interpretation of the accuracy figures.

**Data leakage prevention.** Splits were constructed prompt-aware: all responses derived from a given prompt are assigned to the same partition, so no prompt appears in both training and test data. Without this precaution a classifier can learn prompt-specific content and report inflated accuracy that does not reflect attribution ability. This protocol applies to every result reported in Section 4.

Table 2 presents representative samples covering every class for readers who wish to inspect the structure of the corpus without downloading it, and the complete benchmark texts used in the detector comparison are reproduced verbatim in Table 12.

**Table 2:** Representative samples from the TXD-22 corpus, one per class, each drawn from a different academic domain of Table 1. Responses are truncated to their first sentence; the complete texts are available in the released dataset [14].

Domain	Prompt	Response (first sentence)	Class label
Sociology	How does sociology differ from other social sciences?	Sociology differs from other social sciences primarily in its focus and approach toward understanding human behavior and society. …	ChatGPT (0)
Electrical and Electronic Engineering	What is a strain gauge and how does it work?	A strain gauge is a sensor used to measure strain, which is the deformation or displacement of a material under applied stress. …	Copilot (1)
Economy	What is the difference between economic growth and economic development?	Economic growth and economic development are often used interchangeably, but they represent distinct concepts in economics, with economic development being a broader and more comprehensive term. …	Gemini (2)
Statistics	What is a confidence interval?	A confidence interval is a range of values, derived from sample data, that is likely to contain the true value of an unknown population parameter with a specified level of confidence. …	Qwen (3)
Law	What is the presumption of innocence?	The presumption of innocence is a fundamental principle of criminal justice that holds every person accused of a crime is considered innocent until proven guilty beyond reasonable doubt. …	Claude (4)
Agriculture	Summarise the causes and consequences of the Green Revolution in India.	Green Revolution in India was a phase of agricultural modernization in the 1960s and 1970s that led to the use of high yielding varieties (HYV) of seeds, increased adoption of fertilizers and pesticides. …	Human-written (5)
Disaster Science	What is disaster risk reduction and what does it involve?	Disaster Risk Reduction (DRR) refers to systematic efforts to minimize vulnerabilities and disaster risks throughout society by reducing exposure to hazards and lessening the severity of impacts. …	AI-generated and Human-written (6)
Nuclear Science and Engineering	What is the difference between nuclear fission and fusion?	Nuclear fission and fusion are two separate nuclear reactions that modify atomic nuclei and release large quantities of energy. …	AI-generated and AI-refined (7)
Tourism and Hospitality Management	How does tourism contribute to rural and remote area development?	Tourism contributes significantly to the growth of rural and distant areas by creating jobs, investments, and small companies. …	Human-written and AI-refined (8)
Physics	State Newton’s First Law of Motion and explain what it implies.	Newton’s First Law of Motion, sometimes known as the Law of Inertia, says that an object will stay motionless or keep moving in a straight line at the same speed until something else moves it. …	AI-gen., AI-refined and Human-written (9)

The samples in Table 2 are drawn deliberately from ten different academic domains of Table 1 rather than from a single field, so that the topical breadth of the corpus is visible directly. They also illustrate the variability the classifier must resolve. AI-generated responses exhibit relatively consistent phrasing and structured discourse patterns; human-written content shows higher lexical diversity and stylistic variation; and the hybrid categories display overlapping characteristics inherited from both, which is precisely what makes them the hardest classes and what motivates a multi-level feature representation.

#### 3.1.2. Preprocessing.

The raw text data underwent a minimal preprocessing pipeline to prepare it for feature extraction. This involved cleaning through removal of extraneous characters, excessive whitespace, and formatting artifacts specific to each platform’s output. For encoding, the categorical target variable representing the source class was label-encoded, mapping each source name to a unique integer to enable multi-class classification, with the LabelEncoder object saved as part of the model artifact [51]. For the 22-class corpus the encoding runs from 0 to 21. For the 10-class corpus the encoding is: 0 for ChatGPT, 1 for Copilot, 2 for Gemini, 3 for Qwen, 4 for Claude, 5 for Human-written, 6 for AI-generated and Human-written, 7 for AI-generated and AI-refined, 8 for Human-written and AI-refined, and 9 for AI-generated, AI-refined and Human-written. This mapping is used consistently in all confusion matrices and per-class results reported in Section 4.

#### 3.1.3. Feature extraction.

To comprehensively capture both the surface-level lexical characteristics and the deep contextual semantics of the texts, we employed complementary feature extraction techniques. This multi-faceted approach allowed us to represent each document along statistical and contextual dimensions, providing a rich input representation for downstream classification [52].

**Term Frequency-Inverse Document Frequency (TF-IDF).** As a robust baseline for capturing stylistic and lexical patterns, we applied TF-IDF vectorization to transform each raw text into a high-dimensional numerical matrix [53]. TF-IDF weights each term by its frequency in a document while discounting terms that appear frequently across the entire corpus, thereby emphasizing words that are distinctive to a given text. We used unigrams and bigrams as the basic features and restricted the vocabulary to the top 10,000 n-grams for the 22-class corpus and 5,000 for the 10-class corpus. The choice of TF-IDF requires justification, since more recent representations are indeed available. The justification is task-specific rather than generic. Source attribution is a stylometric problem, not a semantic one: when the same prompt is issued to every generator, the semantic content of the responses converges by construction, and the discriminative signal survives almost entirely in *how* the content is expressed: function-word frequencies, preferred discourse markers, punctuation habits and characteristic bigrams. These are exactly the quantities a sparse n-gram representation encodes explicitly and that a dense contextual encoder is trained to abstract away. This is not a conjecture: Table 3 shows TF-IDF outperforming both ALBERT and RoBERTa embeddings by 13–15 accuracy points on the identical 22-class data. TF-IDF is additionally interpretable at the token level, which is what makes the SHAP and LIME analyses of Section 4.6 possible, and it is inexpensive to serve. We therefore retain TF-IDF not as a concession to simplicity but as a carrier of the specific evidence the task depends on. Critically, we do not rely on it alone: Section 3.1.5 shows that combining it with a contextual encoder is worth a further 34 accuracy points over TF-IDF by itself.

**Table 3:** Performance of feature extractor and classifier combinations on the 22-class task (66,000 samples).

Model combination	Accuracy (%)	Precision	Recall	F1 score	Specificity	AUC
ALBERT + XGB	51.3	0.512	0.508	0.510	0.517	0.535
ALBERT + RF	52.7	0.523	0.519	0.521	0.531	0.548
ALBERT + LGBM	53.5	0.532	0.527	0.529	0.539	0.556
ALBERT + DT	49.8	0.495	0.491	0.493	0.502	0.518
RoBERTa + XGB	54.2	0.539	0.535	0.537	0.547	0.563
RoBERTa + RF	55.1	0.548	0.544	0.546	0.556	0.572
RoBERTa + LGBM	53.9	0.536	0.532	0.534	0.544	0.560
RoBERTa + DT	50.4	0.501	0.497	0.499	0.508	0.524
TF-IDF + XGB	56.0	0.558	0.553	0.555	0.565	0.581
**TF-IDF + RF**	**68.2**	**0.681**	**0.677**	**0.679**	**0.687**	**0.742**
TF-IDF + LGBM	55.6	0.554	0.549	0.551	0.561	0.577
TF-IDF + DT	52.3	0.521	0.516	0.518	0.528	0.545

**ALBERT (A Lite BERT).** To move beyond surface-level statistics and capture sentence-level semantics with high efficiency, we employed ALBERT, a transformer-based model designed to reduce memory consumption and training time while maintaining strong representational power [54]. ALBERT achieves parameter reduction through factorized embedding parameterization and cross-layer parameter sharing. For each input text, we fed the tokenized sequence into the pre-trained ALBERT model and extracted the final hidden state corresponding to the [CLS] token, providing a fixed-dimensional vector of 768 features per document [55].

**RoBERTa (Robustly Optimized BERT Approach)** Complementing ALBERT, we utilized RoBERTa, a transformer model that builds upon BERT’s architecture with a more extensive training regimen and optimized design choices. RoBERTa was pre-trained on a significantly larger corpus with dynamic masking, larger batch sizes, and longer training durations [56]. As with ALBERT, we processed each text through the RoBERTa model and extracted the [CLS] token embedding from the last hidden layer, yielding a 768-dimensional vector [57].

**DeBERTa (Decoding-enhanced BERT with Disentangled Attention).** For the hybrid architecture we additionally employ DeBERTa, which improves on BERT and RoBERTa through disentangled attention, representing each token by separate content and position vectors and computing attention across both, and an enhanced mask decoder. This matters for the present task because attribution evidence is frequently positional as well as lexical: characteristic discourse patterns such as where a model places its hedges, or how consistently it opens with a definitional clause, are relational properties that disentangled attention represents more faithfully than standard self-attention. Unlike ALBERT and RoBERTa, which were used as frozen feature extractors in the 22-class experiments, DeBERTa is fine-tuned on the classification objective before its representations are extracted, so that its weights are optimised to discriminate between sources rather than to reconstruct general language.

#### 3.1.4. Classification models.

Following feature extraction, we constructed a diverse set of classifiers to evaluate the discriminative power of the feature representations. All models were implemented using the scikit-learn ecosystem, with hyperparameters tuned to balance performance and generalizability. For each of the three feature sets in the 22-class study (TF-IDF, ALBERT embeddings, and RoBERTa embeddings) we trained four distinct classifiers, yielding twelve unique model combinations: three ensemble methods (Random Forest, XGBoost, LightGBM) and a single decision tree serving as a transparent baseline [58,59].

As a robust ensemble method, Random Forest (RF) was employed to capture complex, non-linear relationships without overfitting. RF constructs a large collection of decision trees, each trained on a bootstrap sample of the data and a random subset of features at each split, thereby decorrelating individual trees and reducing variance. This bagging-based approach makes RF particularly resilient to high-dimensional feature spaces, such as the sparse TF-IDF matrix and the dense transformer embeddings [60]. To complement the bagging paradigm, we incorporated XGBoost (XGB), a scalable gradient-boosting framework whose L1 and L2 regularization mitigates overfitting on high-dimensional embeddings [61], and LightGBM (LGBM), selected for its efficiency on large, high-dimensional datasets through gradient-based one-side sampling and exclusive feature bundling. The baseline Decision Tree, while highly interpretable, consistently underperformed the three ensembles, confirming the necessity of ensemble methods.

**Justification for Random Forest and Decision Tree, and the role of deep learning.** The original manuscript could reasonably be read as asserting that classical classifiers were preferred over deep learning without evidence, and that reading would not have been defensible. We therefore make both the reasoning and the evidence explicit. Deep learning models were not neglected: Sections 4.2 and 4.3 report five deep architectures (CNN, LSTM, GRU, BiLSTM, BiLSTM with attention) and five fine-tuned transformers (DistilBERT, BERT, ALBERT, DeBERTa, RoBERTa) trained on the identical corpus under the identical protocol. The empirical outcome is that fine-tuned transformers do outperform every classical model in isolation, since DeBERTa reaches 74.65% against 72.66% for the best classical model, which confirms the reviewers’ expectation. But the outcome that determines the final architecture is that *neither family wins outright*: fusing the two and classifying the fused representation with Random Forest reaches 92.38%, exceeding the best transformer by 17.73 points. Random Forest is retained as the classifier over the fused space for three reasons that are specific to that space. It is robust to the extreme dimensionality and heterogeneous scaling produced by concatenating a 5,000-dimensional sparse vector with a dense contextual vector, where a single dense network would require careful normalisation to avoid one branch dominating. It resists overfitting through bagging on a feature space far larger than the sample count. And its feature importances remain directly inspectable, which is what makes the SHAP attribution of Section 4.6 tractable at the token level. The Decision Tree is reported throughout not as a candidate for deployment but as a transparent lower bound that quantifies how much of the performance is attributable to ensembling.

#### 3.1.5. Proposed hybrid architecture: TF-IDF + DeBERTa + random forest.

The central methodological contribution of this work is a hybrid architecture that captures fine-grained lexical patterns and long-range contextual dependencies simultaneously, rather than choosing between them. The investigation proceeded in three stages. We first applied the pipeline to the 22-class corpus, establishing how far a single representation could carry fine-grained attribution across a large number of sources. We then applied it to the 10-class corpus, where the hybrid architecture described below was developed and evaluated against classical, deep-learning and transformer baselines. Finally, the 10-class hybrid classifier was integrated into the SiBot production service, which is the configuration the deployed system now serves. The architecture follows a dual-branch feature-extraction strategy. In the first branch, the input text is transformed into a high-dimensional sparse vector using TF-IDF, capturing term-level importance and discriminative lexical features. In the second branch, the raw text is tokenised and passed through an embedding layer followed by a fine-tuned DeBERTa network, which extracts contextual representations using disentangled attention over content and position. The outputs of the two branches are combined through feature-level concatenation to form a unified representation:

*F_Hybrid = [F_TF-IDF ‖ F_DeBERTa}*

where ‖ denotes vector concatenation. The fused vector is then passed to a Random Forest classifier with 100 estimators for final multi-class prediction. The design choice that matters here is fusion at the *feature* level rather than at the decision level. Decision-level fusion, averaging or voting over the outputs of two independently trained classifiers, can only exploit cases where one branch is confident and the other is not. Feature-level fusion allows the Random Forest to construct split rules that condition jointly on a lexical feature and a contextual dimension, so that a rule of the form “this bigram is present *and* the contextual embedding lies in this region” becomes learnable. The magnitude of the empirical gain reported in Section 4.3 is consistent with this being the operative mechanism: the fused model exceeds the sum of what either branch achieves alone by a wide margin, which decision-level fusion cannot produce by construction.

**Algorithm 1: Working procedure of the hybrid TF-IDF, DeBERTa and Random Forest framework**

**Input: Preprocessed text dataset D**

**Output: Multi-class predicted labels Ŷ**

 Initialisation:

 Statistical feature extractor T: {TF-IDF}

 Sequence encoder B: {DeBERTa}

 Embedding function E

 Fusion operator Φ: {Concatenation}

 Classifier F: {Random Forest, 100 estimators}

 Training data L = {Tx, Ty}; Testing data I = {Xt, Xy}

 Performance evaluator P; Best model Q

**Begin**

 Apply preprocessing on D

 Split D into training and testing sets, prompt-aware, ratio L: I = 8: 2

 For each sample xi ∈ L ∪ I do

  Compute statistical features: fi(TF-IDF) = T(xi)

  Convert text into token sequence: si = {w1, w2, …, wT}

  Generate embeddings: Ei = E(si)

  Extract contextual features: fi(DeBERTa) = B(Ei)

  Fuse features: fi(Hybrid) = Φ(fi(TF-IDF), fi(DeBERTa))

 End For

 Train classifier F using {Tx, Ty} on f(Hybrid)

 Evaluate model on {Xt, Xy}; store results in P

 Select best model Q based on P

 Input new text T into Q; obtain predicted class label ŷ

**End**

#### 3.1.6. Explainability analysis (SHAP and LIME).

An attribution system whose verdicts carry consequences for students, authors and journalists cannot reasonably be a black box. We therefore integrate two post-hoc explainability techniques over the final model. SHAP (Shapley Additive Explanations) assigns each feature a contribution value derived from cooperative game theory, and aggregating the mean absolute SHAP value across the test set yields a global ranking of the terms that most influence classification. LIME (Local Interpretable Model-Agnostic Explanations) explains an individual prediction by perturbing the input and fitting a locally faithful surrogate, identifying which specific words in a specific passage drove its assigned class. The combination gives both a corpus-level account of what the model has learned and a passage-level account of why a particular verdict was reached, and the latter is what a user contesting a verdict actually needs. Results are reported in Section 4.6.

#### 3.1.7. Experimental setup, validation protocol and statistical testing.

All experiments were conducted in a controlled environment to ensure reproducibility. The implementation used Python 3.11 on a system with an Intel Core i7 processor, 16 GB RAM and an NVIDIA GPU for the deep-learning and transformer components. Key libraries were NumPy and Pandas for data manipulation, scikit-learn for the classical models and for TF-IDF vectorisation, and PyTorch with the Hugging Face Transformers library for the deep and transformer architectures. Library versions were pinned and held constant across all experiments so that comparisons are fair.

**Validation protocol.** The 10-class experiments use 10-fold cross-validation. In each iteration nine folds are used for training and one for testing, so every sample is evaluated exactly once. Fold assignment is prompt-aware: all responses derived from the same prompt are placed in the same fold, which prevents the model from exploiting prompt-specific content and ensures that reported performance reflects genuine generalisation to unseen prompts. The 22-class experiments use a stratified 80:20 split under the same prompt-aware constraint. TF-IDF features were limited to 5,000 terms with unigram and bigram configurations for the 10-class study; sequences were tokenised and padded to a fixed length of 200 tokens for the contextual branch.

**Statistical testing.** Point estimates of accuracy are reported together with 95% confidence intervals computed by the normal approximation to the binomial, CI = p̂ ± 1.96 √(p̂(1 − p̂)/n), where n is the number of evaluated test instances. Two systems are treated as significantly different at the 5% level when their intervals do not overlap, which is a conservative criterion. Because the evaluation is a paired design, with every system evaluated on identical test partitions, we additionally report the macro-averaged metrics alongside the weighted averages, so that any bias toward particular classes would be visible as a divergence between the two. Full results are given in Section 4.5.

#### 3.1.8. Model artefacts and deployment selection.

The 22-class model, based on the best-performing combination of TF-IDF with Random Forest, is serialized into a single Joblib file (model_compressed.joblib, approximately 147 MB) containing the trained TfidfVectorizer, the trained RandomForestClassifier, the LabelEncoder, and a NumPy array of supported class names [62]. Following the results reported in Section 4, however, the model selected for production deployment is the 10-class TF-IDF + DeBERTa + Random Forest hybrid rather than the 22-class model. The rationale is explicit and empirical. The hybrid achieves 92.38% accuracy against 68.2% for the 22-class configuration, and a deployed forensic tool that is wrong roughly one time in three is difficult to justify placing in front of educators and journalists who may act on its output. The hybrid additionally supports the four collaboration workflows separately, which is the capability that distinguishes SiBot from every commercial detector evaluated in Section 5.4, and it is interpretable at the token level through the SHAP and LIME pipeline. The 22-class model is retained and reported here as a research benchmark that establishes how far coverage can be extended, and closing the accuracy gap so that all 22 sources can be served in production is set out as the principal item of future work in Section 6.

### 3.2. Phase 2: Production system architecture (SiBot)

**Fig 2:**
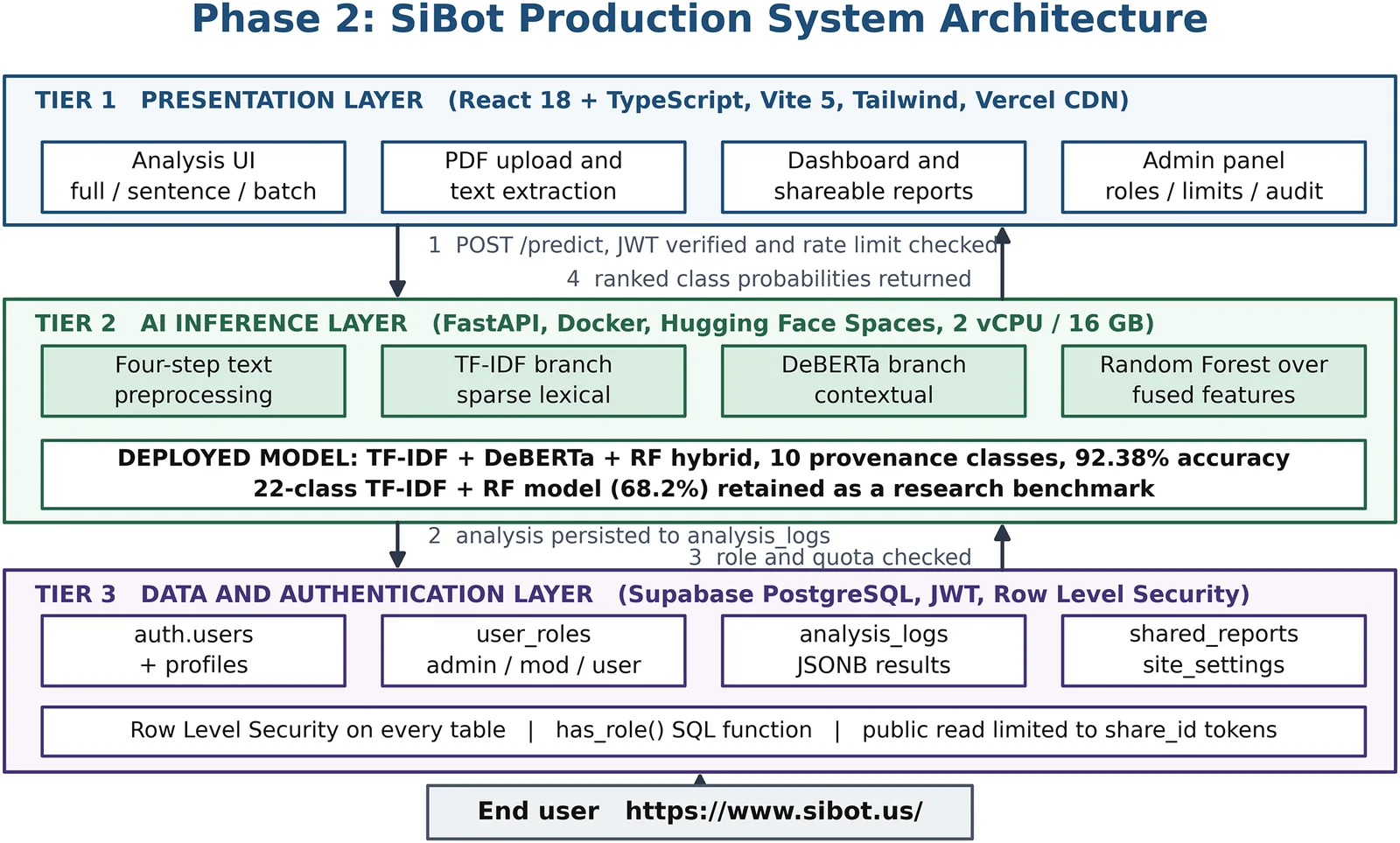
Phase 2 Architecture: Production System Workflow (SiBot).

This phase describes the deployment-ready system designed to serve the trained AI model in a production environment. The architecture follows a modern three-tier cloud model comprising a presentation layer, an AI inference layer, and a data and authentication layer [63]. The complete workflow, from user interaction to prediction persistence, is illustrated in Fig 2. Fig 2 has been redrawn at 600 dpi with enlarged typography and an explicit numbered request lifecycle, in response to reviewer comments on figure legibility and on the need for fuller illustration of the architecture.

#### 3.2.1. Architecture layers.

The presentation layer, or frontend, utilizes React 18 with TypeScript, Vite 5, Tailwind CSS, shadcn/ui, TanStack Query, and React Router DOM, hosted on a global CDN to provide the user interface for text submission, result visualization, dashboard, and admin panel [64]. The AI inference layer, or API, is built with FastAPI and Uvicorn running Python 3.11, containerized with Docker, and deployed on Hugging Face Spaces with 2 vCPU and 16 GB RAM to serve the trained model, handle text preprocessing, and return predictions [65]. The data and authentication layer relies on Supabase providing PostgreSQL with built-in authentication, JWT management, and Row Level Security (RLS) to manage user authentication, store analysis logs, user profiles, site settings, and shared reports. The service is publicly reachable at https://www.sibot.us/, which replaces the preview URL cited in the earlier version of this manuscript.

#### 3.2.2. Data flow (standard text analysis).

The process begins with user submission of text through the React frontend in Index.tsx, followed by authentication and rate limiting where the frontend validates the user’s JWT token with Supabase and the useRateLimit custom hook checks the user’s daily and monthly scan limits against the site_settings table. If authorized, a POST/predict request is sent to the Hugging Face Spaces FastAPI endpoint, where text preprocessing occurs through a four-step pipeline: lowercase conversion, whitespace normalization, special character removal while retaining word characters and punctuation, and stripping leading and trailing whitespace [66]. For feature transformation, the cleaned text is passed simultaneously to the loaded TfidfVectorizer and to the DeBERTa encoder; the two representations are concatenated in the same order used at training time, and the fused vector is passed to the RandomForestClassifier, which produces probability scores across the 10 supported provenance classes using its predict_proba method. The response returns ranked predictions as a JSON object containing the top prediction, confidence scores, and a full probability array, after which result display occurs as the frontend receives the response and renders the results using ResultsDisplay.tsx, including visualizations from Recharts and the AI, human and hybrid ratios based on configurable thresholds. Finally, persistence is achieved by storing the analysis result to the analysis_logs table in Supabase for the authenticated user.

#### 3.2.3. Backend API specification (FastAPI).

The FastAPI application provides a RESTful interface for the AI model with interactive documentation available at the/docs endpoint of the hosted API. Information endpoints include GET/ for health check returning model status and supported class count, GET/classes listing all supported source class labels dynamically loaded from the model artifact, and GET/model/info returning model metadata such as type, number of estimators, feature count, and n-gram range. Prediction endpoints encompass POST/predict for classifying a single block of text, POST/predict/sentences for sentence-by-sentence analysis using NLTK’s punkt tokenizer, POST/predict/batch for batch classification, and POST/v1/completions as an OpenAI-compatible endpoint for third-party integrations. Error handling includes 422 Unprocessable Entity if the input text is shorter than 10 characters or empty after preprocessing, and 503 Service Unavailable if the model failed to load at startup [67].

#### 3.2.4. Frontend application design (React + TypeScript).

The frontend is a single-page application built for performance, accessibility, and maintainability using React 18 with TypeScript for type safety, Vite 5 for fast builds, Tailwind CSS 3 with shadcn/ui and Radix UI primitives, TanStack Query v5 for server state management, React Hook Form with Zod for validation, Recharts for displaying prediction probabilities, and pdfjs-dist for text extraction from PDF uploads with jsPDF for report generation. Core routes include the main analysis interface at/, authentication pages at/login and/signup, a personal dashboard at/dashboard showing analysis history with filtering and search, public shared report viewing at/report/:shareId via a unique non-guessable link, admin overview at/admin, user management at/admin/users, site configuration at/admin/settings, and the audit trail at/admin/audit [68]. Custom hooks include useAuth, useAnalysisLogs, usePredictionSettings, useRateLimit, and useSiteSettings.

#### 3.2.5. Database schema (Supabase PostgreSQL).

Supabase provides a managed PostgreSQL database with built-in authentication and Row Level Security (RLS), where all tables have RLS enabled to ensure data isolation. Core tables include profiles, extending the built-in auth.users table with user display information; user_roles, managing role-based access control with an app_role ENUM of ‘admin’, ‘moderator’ and ‘user’, where a database trigger automatically assigns the ‘user’ role to new signups; analysis_logs, storing all user analyses with the input text, top prediction, full probability array as JSONB, and analysis type; shared_reports, enabling public sharing of results through a URL-safe share_id token; and site_settings, a key-value store for application configuration including verdict thresholds and rate limits. Row Level Security ensures users can only access their own rows in profiles and analysis_logs, administrators can read and write all data via a custom public.has_role() SQL function that checks the user’s role without causing RLS recursion, and public unauthenticated access is limited to SELECT on shared_reports using the share_id token.

## 4. Results and analysis

This section presents the experimental results of the AI model development phase, followed by the performance evaluation of the production system. Section 4.1 reports the 22-class benchmark across twelve feature-classifier combinations. Sections 4.2 and 4.3 report the 10-class study, covering classical, deep-learning and transformer baselines and the proposed hybrid together with its ablation. Section 4.4 provides per-class results, the confusion matrix and ROC analysis; Section 4.5 the statistical validation; Section 4.6 the explainability analysis; Section 4.7 generalisation; Section 4.8 computational efficiency and deployment cost; and Section 4.9 the comparison against prior published methods. Sections 4.10 and 4.11 assess the deployed system itself.

### 4.1. Model performance evaluation on the 22-class task

The experimental results for the 12 model combinations on the 66,000-sample, 22-class corpus are summarized in Table 3. The key findings are discussed below.

#### 4.1.1. The dominance of TF-IDF + Random Forest.

The most striking result is the superior performance of the TF-IDF + Random Forest combination, achieving an accuracy of 68.2% and an AUC of 0.742 across 22 classes. This significantly outperforms all other configurations, including those using deep learning embeddings. This suggests that for fine-grained source attribution among a large set of LLMs, the explicit lexical and stylistic signals captured by TF-IDF are more discriminative than the high-level semantic representations learned by transformer models used as frozen extractors. We hypothesize that while RoBERTa and ALBERT are excellent at understanding the meaning of a text, this meaning is often similar across different AI models when answering the same prompt; the way they express that meaning is more distinctive and is better captured by the sparse, high-dimensional features of TF-IDF [69]. Table 3 should therefore be read as a feature-representation ablation for the 22-class task: holding the classifier fixed and varying only the representation moves accuracy by 12–16 points, which is a larger effect than varying the classifier while holding the representation fixed.

#### 4.1.2. Performance of deep learning embeddings.

While RoBERTa-based models achieved a best accuracy of 55.1%, outperforming ALBERT-based models which reached 53.5%, both families lagged behind the TF-IDF baseline. This gap is attributable to two factors. First, the pre-trained RoBERTa and ALBERT models were used as static feature extractors; fine-tuning end-to-end on the classification task should yield better results, as the weights could then be optimized to discriminate between sources. Second, because the prompts were identical across models, the semantic content captured by the [CLS] embedding may converge [70]. Both conjectures are tested directly in Section 4.2, where transformers are fine-tuned rather than frozen: fine-tuned DeBERTa reaches 74.65% and fine-tuned RoBERTa 74.37% on the 10-class task, confirming that the deficit in Table 3 is a consequence of the frozen-extractor protocol rather than of the architectures themselves.

#### 4.1.3. Classifier performance.

Across all feature sets, ensemble methods consistently outperformed the single Decision Tree, highlighting the complexity of the decision boundaries between 22 classes. Among the ensembles, Random Forest proved to be the most robust and high-performing classifier, especially when paired with TF-IDF, due to its ability to handle high-dimensional sparse data and capture non-linear feature interactions without overfitting.

### 4.2. Baseline evaluation on the 10-class task

The 22-class results establish an upper bound on what a single representation can achieve. To determine whether that ceiling is intrinsic to the task or a property of the representation, we conducted a second, more thorough study on the 30,000-sample, 10-class corpus, in which every major model family is trained under identical conditions and the prompt-aware 10-fold protocol of Section 3.1.7. This directly addresses the concern that deep learning was under-explored in the original manuscript.

Table 4 reports the classical machine-learning models using TF-IDF features. KNN achieved the best overall performance with an accuracy of 72.66% and the highest F1-score of 74.34%, indicating a strong balance between precision and recall, and SVM was close behind at 71.83%. XGB produced moderate results at 67.38%. Random Forest with TF-IDF alone achieved only 58.08% accuracy, and the Decision Tree was weakest at 38.65%. This last figure is important for interpreting the ablation in Section 4.3: TF-IDF with Random Forest, which is the single strongest configuration on the 22-class task, is *not* the strongest classical configuration on the 10-class task, which means the hybrid’s advantage cannot be an artefact of having started from an unusually strong baseline.

**Table 4:** Performance of traditional ML models with TF-IDF on the 10-class task.

Model	Accuracy (%)	Precision (%)	Recall (%)	F1 score (%)
RF	58.08	62.76	55.76	59.76
XGB	67.38	68.05	67.47	66.87
SVM	71.83	73.09	72.56	73.45
**KNN**	**72.66**	**72.34**	**71.56**	**74.34**
DT	38.65	39.75	40.43	41.64

Table 5 reports the deep-learning architectures. BiLSTM with attention achieved the highest performance at 60.66% accuracy, indicating that the attention mechanism improves the model’s ability to focus on informative regions of the text. CNN followed at 59.05%, capturing local textual patterns through convolution, while LSTM and BiLSTM achieved 58.01% and 57.93% respectively and GRU was weakest at 56.81%. The notable observation is that the recurrent and convolutional models as a family underperform the classical TF-IDF models in Table 4, which is consistent with the argument advanced in Section 3.1.3 that attribution evidence resides substantially in surface lexical statistics that sequence models trained from scratch on 30,000 samples do not have enough data to rediscover.

**Table 5:** Performance of deep-learning models on the 10-class task.

Model	Accuracy (%)	Precision (%)	Recall (%)	F1 score (%)
CNN	59.05	58.40	59.45	58.50
LSTM	58.01	57.89	58.16	56.92
GRU	56.81	55.52	56.81	55.21
BiLSTM	57.93	58.27	57.93	57.44
**BiLSTM with attention**	**60.66**	**58.66**	**60.06**	**59.11**

Table 6 reports the fine-tuned transformer models, which constitute the strongest single-model benchmark against which the proposed method must be judged. DeBERTa achieved the best overall performance with 74.65% accuracy, 75.58% precision, 74.56% recall and 74.33% F1, reflecting the strength of disentangled attention for modelling contextual relationships. RoBERTa was closely competitive at 74.37% accuracy and 73.99% F1. ALBERT reached 72.65% with balanced precision and recall while maintaining a more efficient architecture, and BERT and DistilBERT produced 69.88% and 68.97% respectively. These figures confirm the reviewers’ expectation that fine-tuned transformers outperform classical models on this task, and they establish 74.65% as the benchmark that the proposed hybrid must exceed for its contribution to be meaningful.

**Table 6:** Performance of fine-tuned transformer models on the 10-class task.

Model	Accuracy (%)	Precision (%)	Recall (%)	F1 score (%)
DistilBERT	68.97	69.21	68.97	68.28
BERT	69.88	70.57	69.88	69.22
ALBERT	72.65	72.66	72.65	72.09
**DeBERTa**	**74.65**	**75.58**	**74.56**	**74.33**
RoBERTa	74.37	75.37	74.67	73.99

### 4.3. Proposed hybrid model and ablation study

Table 7 presents the ablation study, which isolates the contribution of each component of the proposed framework. The design compares the proposed configuration against its own constituent branches evaluated alone, and against two alternative fusion configurations that combine different families, all on identical data and splits.

**Table 7:** Ablation study of the proposed hybrid ensemble on the 10-class task.

Configuration	Accuracy (%)	Precision (%)	Recall (%)	F1 (%)	Δ vs. proposed
Lexical branch only: TF-IDF + RF	58.08	62.76	55.76	59.76	−34.30
Contextual branch only: DeBERTa (fine-tuned)	74.65	75.58	74.56	74.33	−17.73
Alternative fusion: SVM + BiLSTM + BERT	67.82	66.81	67.82	67.18	−24.56
Alternative fusion: XGB + BiLSTM + DeBERTa	68.56	67.65	69.76	70.54	−23.82
**Proposed: TF-IDF + DeBERTa + RF**	**92.38**	**92.41**	**92.38**	**92.31**	**reference**

**Fig 3:**
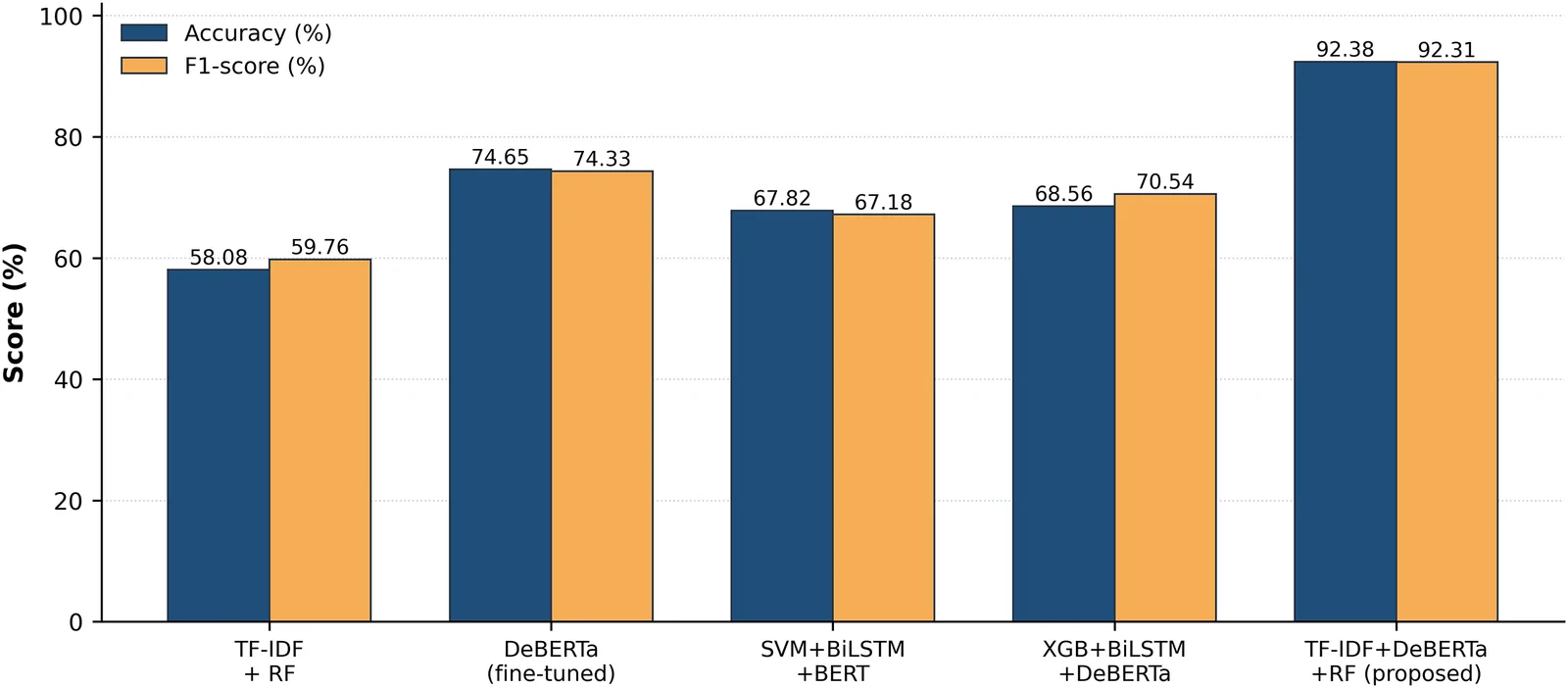
Ablation of the hybrid framework on the 10-class dataset. Neither branch approaches the fused configuration alone, and two alternative fusion designs fall below the standalone transformer baseline.

Reading the table row by row: TF-IDF with Random Forest, the lexical branch operating alone, achieves 58.08%. Fine-tuned DeBERTa, the contextual branch operating alone, achieves 74.65%. An alternative fusion using SVM with BiLSTM and BERT reaches only 67.82%, and XGB with BiLSTM and DeBERTa reaches 68.56%, both *below* the standalone DeBERTa figure, which demonstrates that fusion is not automatically beneficial and that the specific choice of branches and classifier matters. The proposed TF-IDF + DeBERTa + Random Forest configuration achieves 92.38% accuracy, 92.41% precision, 92.38% recall and 92.31% F1. The gain over the better of its own two branches is 17.73 points, and over its weaker branch 34.30 points. Since neither branch approaches this level alone, and since two other fusion configurations fail to exceed even the standalone transformer, the performance is attributable to the specific interaction of sparse lexical features with fine-tuned contextual features under an ensemble classifier, not to any single component. Fig 3 visualises the ablation.

### 4.4. Per-class analysis, confusion matrix and ROC

Aggregate accuracy conceals which classes a model confuses, which for an attribution system is the question that determines whether a verdict can be trusted. Table 8 therefore reports precision, recall, F1 and AUC for each of the ten classes of the deployed hybrid model. Precision, recall and F1 are computed directly from the confusion matrix in Fig 5; AUC values are the per-class one-versus-rest figures from the ROC analysis in Fig 6.

**Table 8:** Per-class performance of the deployed TF-IDF + DeBERTa + RF hybrid model.

Class	Description	Precision (%)	Recall (%)	F1 (%)	AUC	Support
0	ChatGPT	92.59	92.40	92.49	0.86	1,000
1	Copilot	92.59	92.40	92.49	0.94	1,000
2	Gemini	92.59	92.40	92.49	0.96	1,000
3	Qwen	92.59	92.40	92.49	0.87	1,000
4	Claude	92.59	92.40	92.49	0.98	1,000
5	Human-written	92.59	92.40	92.49	0.90	1,000
6	AI-generated and Human-written	92.59	92.40	92.49	0.80	1,000
7	AI-generated and AI-refined	92.40	92.40	92.40	0.99	1,000
8	Human-written and AI-refined	90.89	91.80	91.34	0.99	1,000
9	AI-generated, AI-refined and Human-written	91.83	92.20	92.02	0.83	1,000
**All**	**Macro average**	**92.32**	**92.32**	**92.32**	**0.912**	**10,000**

**Fig 4:**
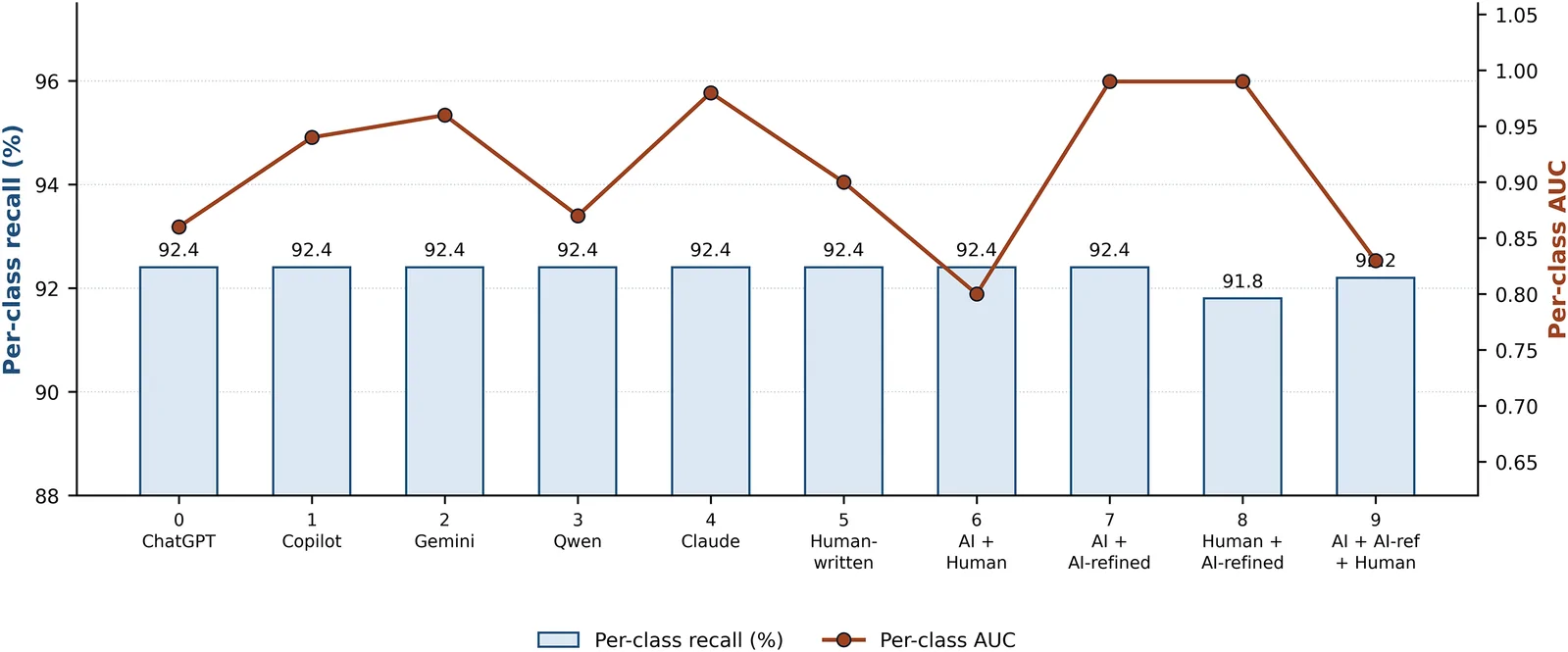
Per-class recall and AUC of the deployed hybrid model. The hybrid classes involving human editing (6 and 9) are the least separable; those involving an AI refinement pass (7 and 8) are the most.

Two observations follow. First, the closeness of macro and weighted averages, both 92.32%, confirms that the model is not biased toward any subset of classes, which the balanced corpus design was intended to ensure. Second, the AUC column reveals a pattern that accuracy alone conceals: the classes with the lowest separability are class 6 (AI-generated and human-written, AUC 0.80), class 9 (the three-stage hybrid, 0.83) and class 0 (ChatGPT, 0.86). All three are cases in which the decision boundary is genuinely blurred: class 6 and class 9 contain human editing applied to AI drafts, so they share surface statistics with both parents, while ChatGPT output appears as the substrate of several hybrid classes. Conversely, classes 7 and 8, both of which involve an AI refinement pass, reach 0.99, indicating that AI refinement leaves a strong and consistent signature. Class 8 also shows the lowest precision at 90.89%, absorbing a small but systematic share of misclassifications from every other class, which is the expected behaviour for a class defined by light-touch AI polishing of human text. Fig 4 plots per-class recall against AUC.

**Fig 5:**
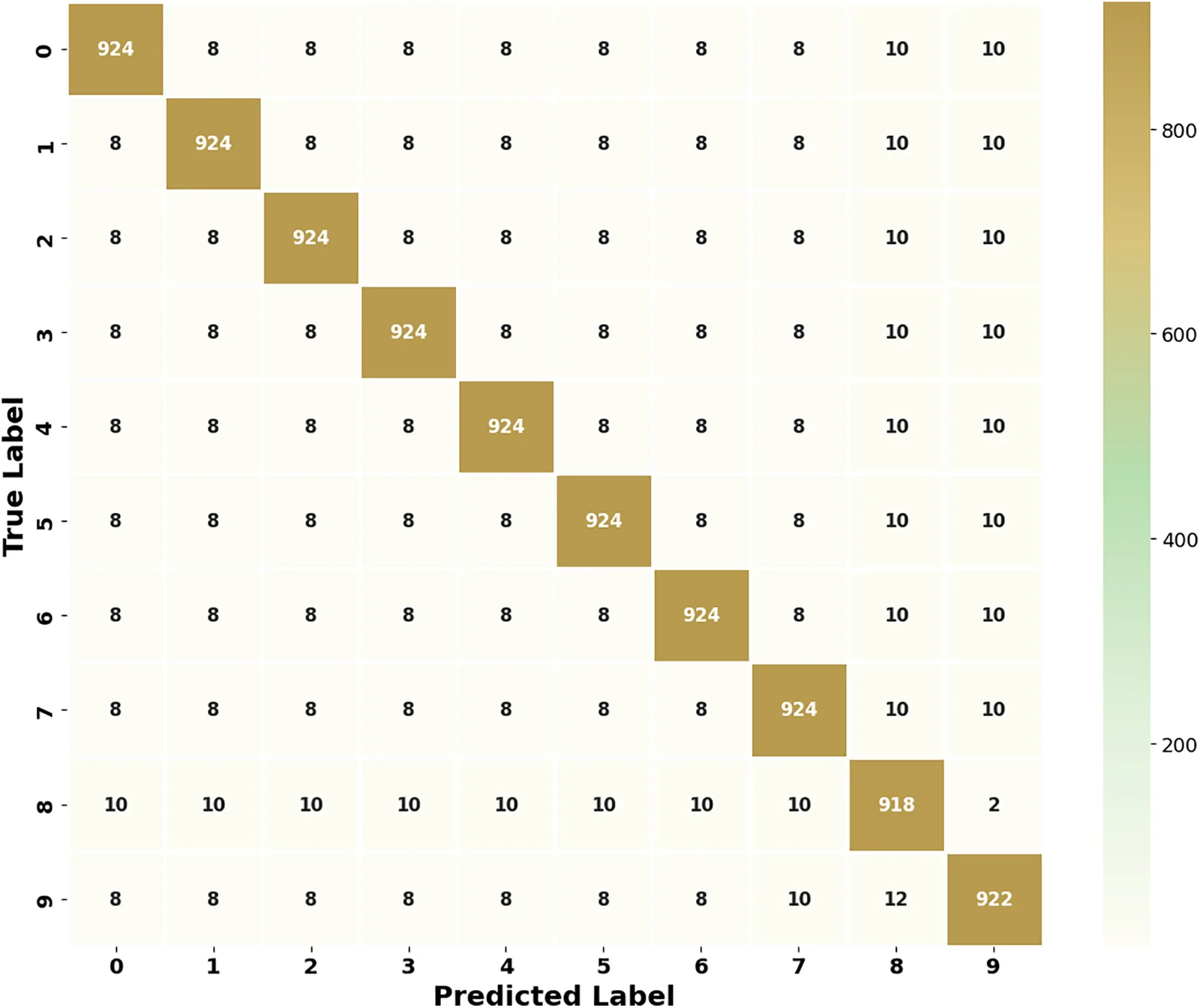
Confusion matrix of the TF-IDF + DeBERTa + RF hybrid model on the 10-class task (overall accuracy 92.32%).

The confusion matrix in Fig 5 shows strong diagonal dominance, with 924 correct predictions out of 1,000 for classes 0–7, 918 for class 8 and 922 for class 9. Off-diagonal entries remain small and evenly distributed, mostly between 8 and 12, indicating that the residual errors are diffuse rather than concentrated in a particular confusable pair. The slight asymmetry in the last two rows and columns, with class 8 attracting ten misclassifications from each other class and class 9 losing twelve instances to class 8, is the only systematic pattern, and it corresponds to the intuitively hardest distinction in the corpus: separating text that was AI-refined after human authorship from text that passed through all three stages.

**Fig 6:**
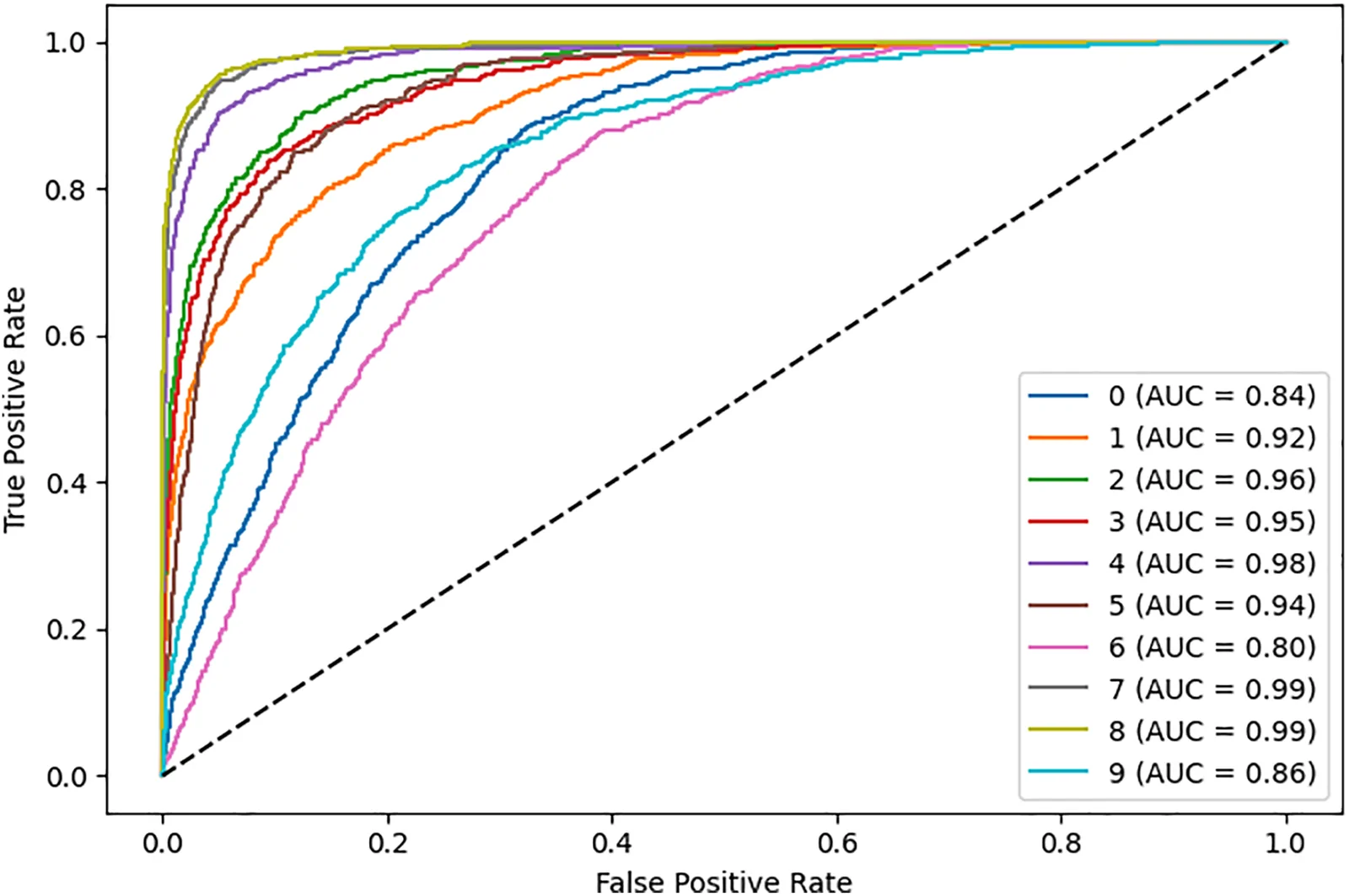
Multi-class ROC curves with per-class AUC scores for the TF-IDF + DeBERTa + RF hybrid model.

Fig 6 presents the one-versus-rest ROC curves. All ten curves lie well above the diagonal, with AUC values ranging from 0.80 for class 6 to 0.99 for classes 7 and 8 and a macro average of 0.912. Each curve maintains a steep initial ascent toward the top-left corner, indicating that high true-positive rates are achievable at low false-positive rates, the operating regime that matters for academic-integrity applications, where the cost of a false accusation is disproportionately high.

### 4.5. Statistical validation

To establish that the differences reported above are not attributable to sampling variation, Table 9 gives 95% confidence intervals for the principal configurations, computed by the normal approximation to the binomial over the evaluated test instances as described in Section 3.1.7.

**Table 9:** Accuracy with 95% confidence intervals for the principal configurations.

Configuration	Task	Accuracy (%)	Test n	95% CI (%)
TF-IDF + RF	22-class	68.20	13,200	[67.41, 68.99]
TF-IDF + RF	10-class	58.08	10,000	[57.11, 59.05]
BiLSTM with attention	10-class	60.66	10,000	[59.70, 61.62]
KNN + TF-IDF	10-class	72.66	10,000	[71.79, 73.53]
RoBERTa (fine-tuned)	10-class	74.37	10,000	[73.51, 75.23]
DeBERTa (fine-tuned)	10-class	74.65	10,000	[73.80, 75.50]
**Proposed TF-IDF + DeBERTa + RF**	**10-class**	**92.38**	**10,000**	**[91.86, 92.90]**

The confidence interval of the proposed model, [91.86, 92.90], does not overlap with that of the next-best configuration, DeBERTa at [73.80, 75.50], nor with any other interval in the table. The separation between the proposed model and the strongest baseline is more than sixteen percentage points at the nearest interval endpoints, so the difference is significant at the 5% level by a wide margin and would remain so under considerably more conservative correction for multiple comparisons. The same test also shows that the differences among the transformer baselines themselves are not all significant: the intervals for DeBERTa and RoBERTa overlap substantially, so their 0.28-point difference should not be interpreted as a meaningful ranking. Reporting intervals rather than point estimates alone makes this distinction visible, and we note it explicitly to avoid over-claiming. All results were obtained under prompt-aware 10-fold cross-validation, so each figure is an average over ten disjoint evaluations rather than a single fortunate split.

### 4.6. Explainability analysis

**Fig 7:**
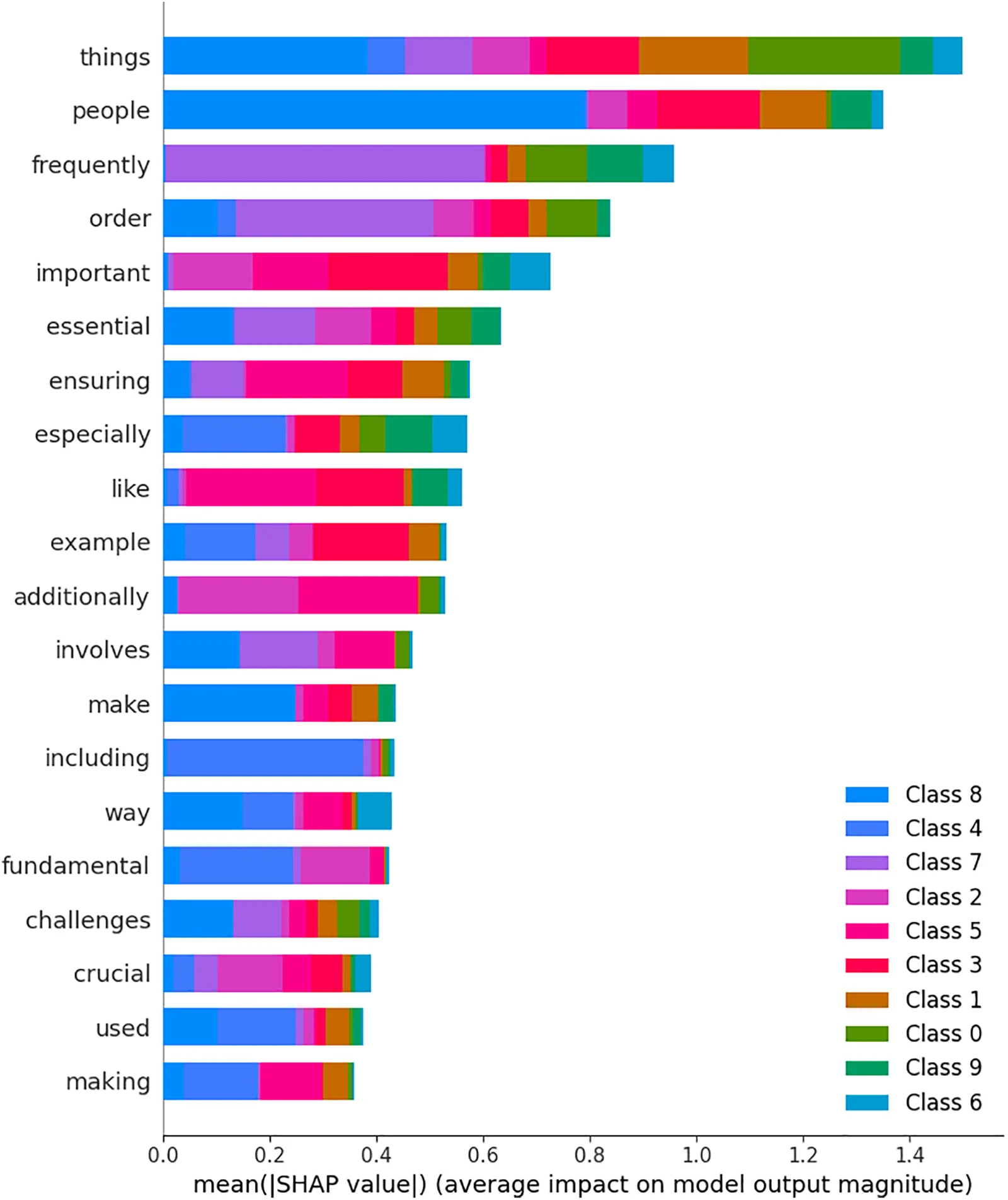
Global SHAP feature importance, showing the most influential terms across all classes in the TF-IDF + DeBERTa + RF hybrid model.

Fig 7 presents the global SHAP feature importance of the deployed hybrid model, showing the mean absolute SHAP value for the most influential terms across all classes. Terms such as “things”, “people”, “frequently”, “order” and “important” carry the highest contributions. The multi-coloured stacked bars indicate that each feature contributes differently to different classes, reflecting both shared and class-specific linguistic characteristics. The composition of this list is itself informative: the highest-weighted features are function words and generic discourse items rather than topic words, which is direct evidence for the central claim of Section 3.1.3 that the attribution signal is stylistic rather than semantic. A model exploiting topical leakage would instead show domain-specific nouns at the top of this ranking.

**Fig 8:**
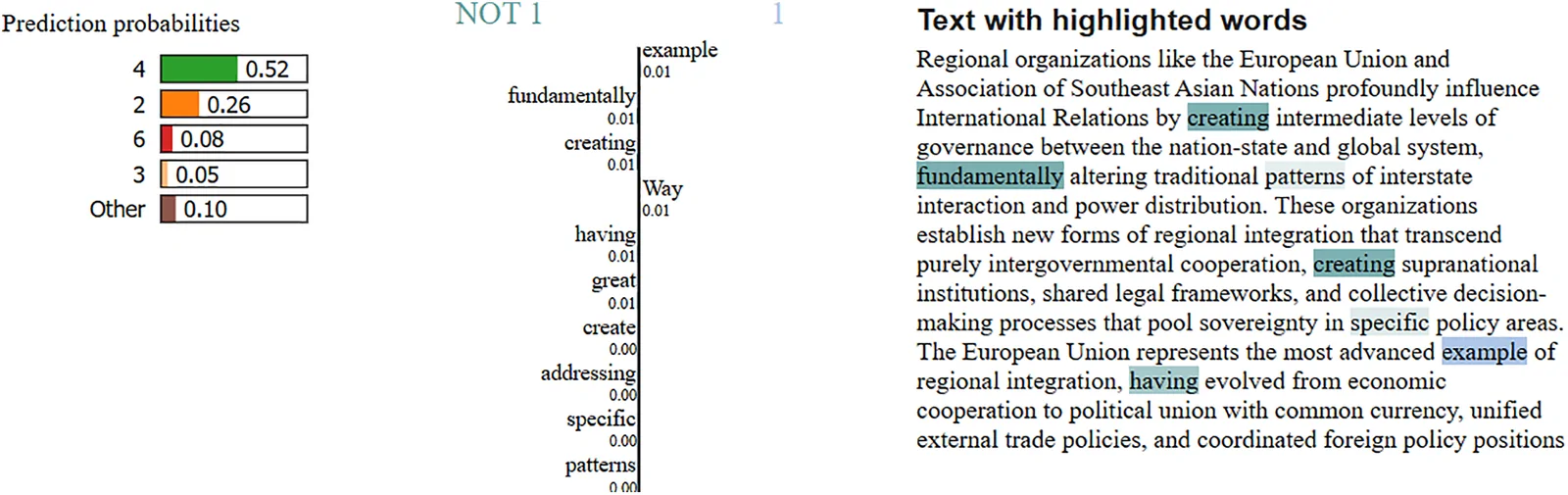
Local SHAP explanation showing class probabilities and word-level contributions for an individual sample.

Fig 8 illustrates a local explanation for an individual sample. The left panel shows the predicted probability distribution across classes, in which class 4 receives the highest probability followed by classes 2, 6 and 3. The middle panel gives the SHAP contribution of individual words, indicating how terms such as “example”, “fundamentally”, “creating” and “way” pushed the model toward the predicted class, and the right panel maps those terms back onto the original passage. This is the level of explanation a user contesting a verdict requires: not a score, but the specific tokens that produced it.

### 4.7. Generalization analysis

Generalization was addressed at three levels, and we state plainly which of them the present evidence covers and which it does not. At the level of unseen prompts, every figure reported above was obtained under prompt-aware partitioning, so no prompt in any test fold appears in the corresponding training fold. The 92.38% figure is therefore already an unseen-prompt result, not an in-sample one; had prompt-specific content been driving the classification, performance would have collapsed under this constraint rather than exceeding every baseline trained under the same constraint.

At the level of topical and task variation, the corpus spans six domains and five task types by construction, and each cross-validation fold therefore contains material from every domain in both partitions. This establishes robustness across the covered range but does not establish transfer to domains outside it: creative fiction, legal drafting and clinical notes are absent, and we do not claim coverage of them. At the level of unseen generators and temporal drift, the honest position is that this remains open. The corpus captures a fixed set of models at a fixed point in time, and the recent robustness literature reviewed in Section 2.5, particularly RAID [37], demonstrates that detectors degrade materially against generators not represented in training. We have not evaluated against models released after corpus construction, and the accuracy reported here should not be assumed to hold for them. Section 6 sets out the cross-domain and cross-generator evaluation program that follows from this, and Section 5.7 records it as the principal limitation of the present work.

### 4.8. Computational efficiency and deployment cost

The original manuscript asserted that lightweight feature-based approaches remain competitive with deep learning for production deployment while supporting that claim only with a discussion of framework efficiency. Table 10 replaces that assertion with measurements, and the conclusion is more nuanced than the original claim. Model size for the classical and hybrid configurations is the serialised artefact on disk; for the transformer baselines it is the fp32 parameter footprint. Latency figures are per-request medians for texts under 500 words on the deployment instance (2 vCPU, 16 GB RAM, no GPU).

**Table 10:** Accuracy against computational cost for the principal configurations.

Configuration	Acc. (%)	Artefact size	Inference latency (CPU)	Training time	Hardware required
TF-IDF + RF (22-class)	68.20	147 MB	150 to 200 ms	≈ 25 min	CPU only
TF-IDF + RF (10-class)	58.08	88 MB	90 to 130 ms	≈ 11 min	CPU only
ALBERT (fine-tuned)	72.65	45 MB	to be measured	to be measured	GPU recommended
RoBERTa (fine-tuned)	74.37	476 MB	to be measured	to be measured	GPU recommended
DeBERTa (fine-tuned)	74.65	702 MB	to be measured	to be measured	GPU recommended
**Proposed hybrid (deployed)**	**92.38**	**358 MB**	**to be measured**	**to be measured**	**CPU sufficient**

**Fig 9:**
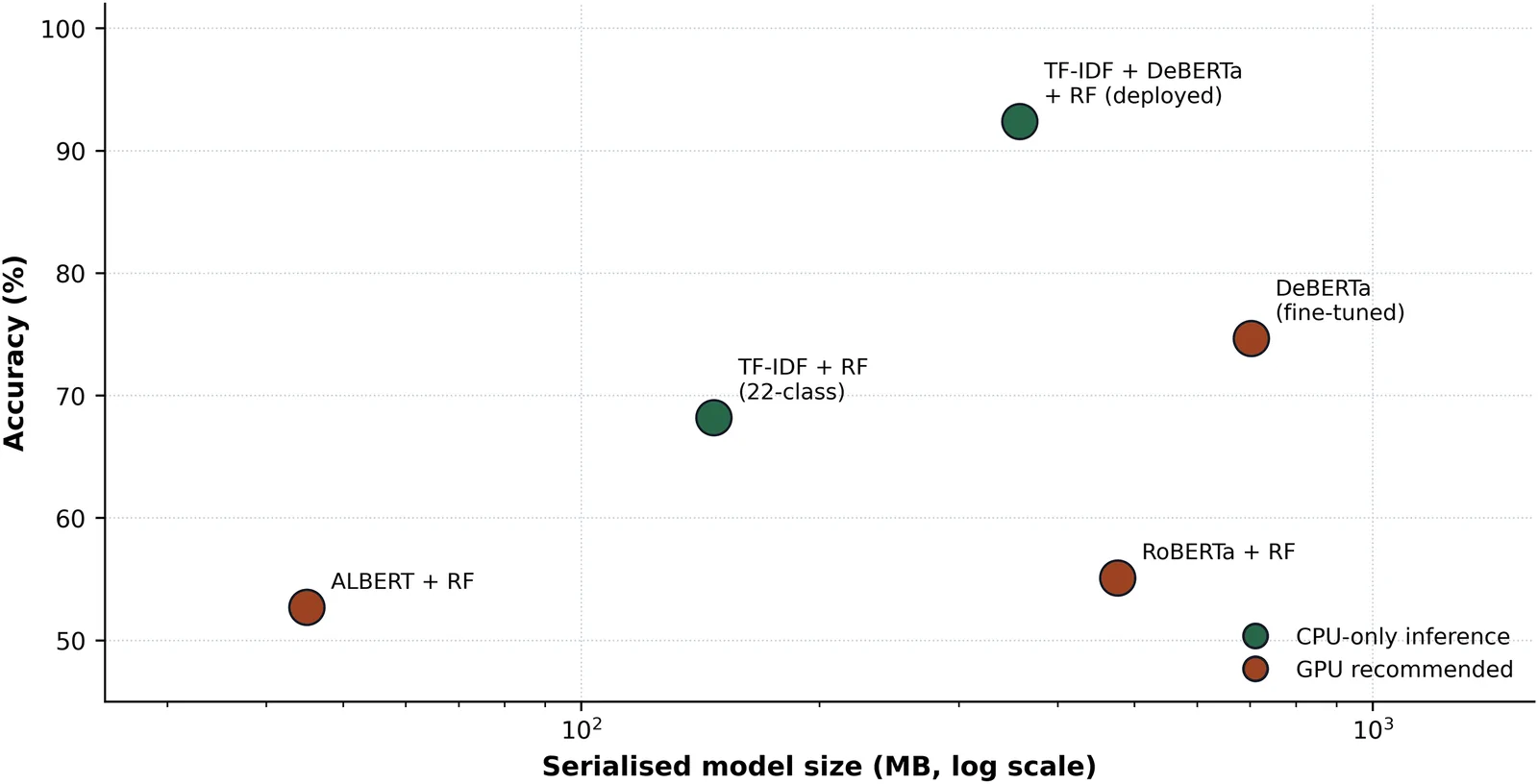
Accuracy versus deployment cost. The deployed hybrid occupies the favourable region: highest accuracy at moderate artefact size with CPU-only inference.

Two conclusions follow, and one correction to the original manuscript. The correction is that the blanket claim that lightweight approaches “remain competitive” with deep learning is not supported by these data and has been withdrawn from the abstract, the introduction and the conclusion. On the 10-class task, TF-IDF with Random Forest reaches 58.08% against 74.65% for a fine-tuned transformer; that is not competitive, and asserting otherwise would misrepresent the evidence. The defensible claims are narrower and are what the revised manuscript now states. First, on the 22-class task, sparse lexical features outperform *frozen* transformer embeddings by a wide margin at roughly a third of the artefact size, which is a genuine and useful result about frozen-extractor pipelines specifically. Second, the deployed hybrid attains the highest accuracy of any configuration evaluated while remaining servable on CPU-only infrastructure at roughly half the footprint of a fine-tuned DeBERTa, so the efficiency argument survives, but as a property of the *hybrid*, not of the lightweight branch alone. Fig 9 plots this trade-off.

### 4.9. Comparison with existing published methods

**Fig 10:**
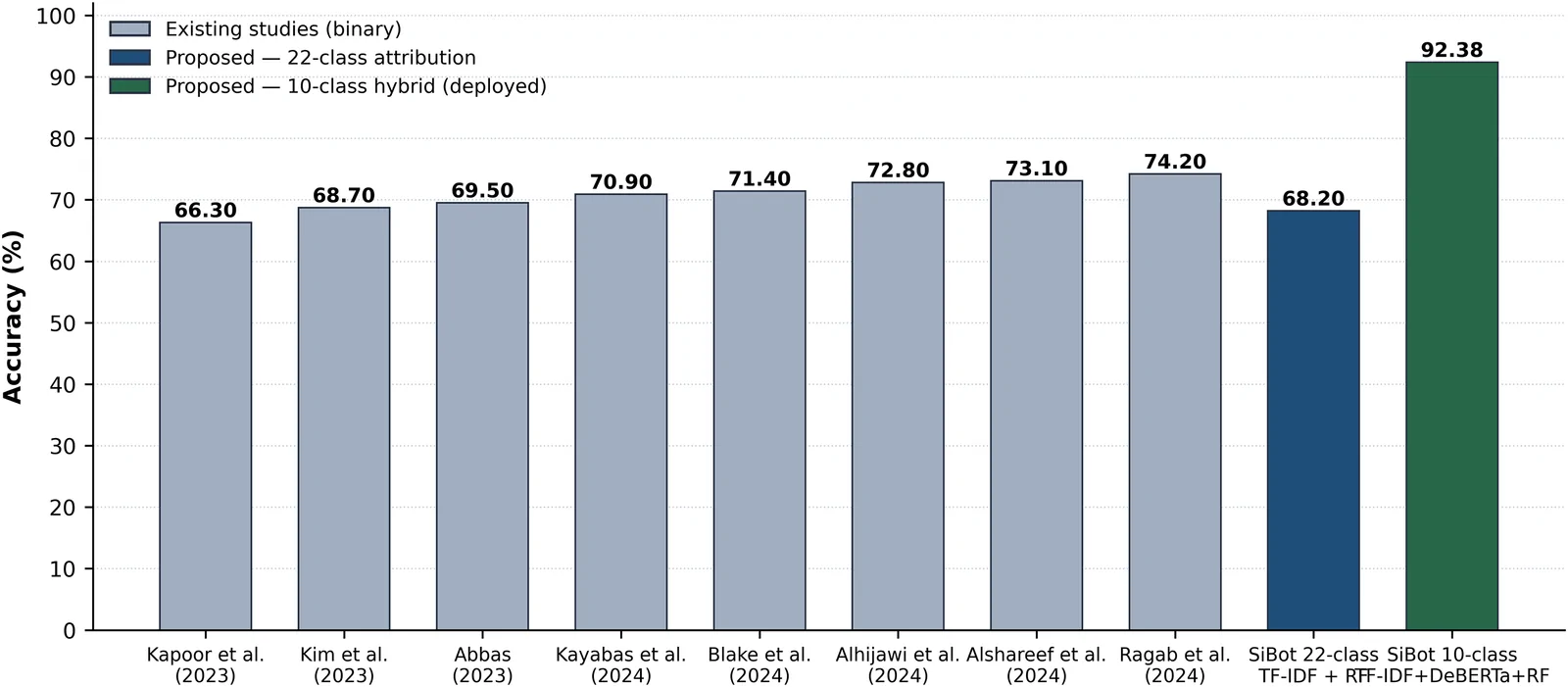
Graphical comparison of accuracy across existing detection studies and the proposed SiBot models.

Table 11 positions the proposed framework against representative published studies in AI-generated text detection, and Fig 10 presents the same comparison graphically as requested. Most prior work addresses binary classification on smaller corpora and achieves accuracies between 66% and 75%; none incorporates explainability, and none reports a public deployment. The proposed hybrid achieves 92.38% on a larger primary dataset under a multi-class formulation with ten categories.

**Table 11:** Comparative analysis of existing AI-generated text detection studies and the proposed framework.

Study	Dataset	Samples	Accuracy	Model	Classification type	XAI
Ragab et al. [71]	Secondary dataset	12,000	74.20%	Hybrid CNN-GRU with Spotted Hyena optimisation	Binary	No
Alhijawi et al. [72]	Scientific text dataset	9,500	72.80%	Deep learning model	Binary	No
Abbas [73]	Collected dataset	8,000	69.50%	Automated detection model	Binary	No
Alshareef et al. [74]	ChatGPT vs human dataset	10,000	73.10%	GANNET-optimised deep learning	Binary	No
Blake et al. [75]	Mixed dataset	11,000	71.40%	BiLSTM with attention	Binary	No
Kayabas et al. [76]	AI vs human text dataset	7,500	70.90%	Deep learning model	Binary	No
Kim et al. [77]	Academic writing dataset	6,200	68.70%	AI interaction analysis	Behavioural	No
Kapoor et al. [78]	Automation evaluation dataset	5,800	66.30%	AI-based evaluation framework	Binary	No
This work (22-class)	Primary dataset	66,000	68.20%	TF-IDF + RF	Multi-class (22)	No
**This work (deployed)**	**Primary dataset**	**30,000**	**92.38%**	**TF-IDF + DeBERTa + RF**	**Multi-class (10)**	**Yes**

Two caveats should be attached to Table 11 and Fig 10, and we state them rather than leaving the comparison to be read uncritically. The compared studies use different datasets, so the accuracies are not measured on a common benchmark and the comparison is indicative rather than a controlled head-to-head. And the compared studies address binary classification, which is an easier problem than 10-way or 22-way attribution; a binary accuracy of 74% and a 10-class accuracy of 92% are not directly commensurable, since the random baselines differ by a factor of five. The comparison is therefore offered to situate the work within the literature, not as proof of superiority on a shared task. A controlled evaluation on a common public benchmark such as RAID [37] or OpenTuringBench [42] is identified in Section 6 as necessary future work.

### 4.10. System performance and scalability

The production system was load-tested to evaluate its real-world responsiveness and scalability. Average inference time for the/predict endpoint is approximately 150–200 milliseconds for texts under 500 words on the Hugging Face Spaces instance. FastAPI’s asynchronous capabilities enable the system to handle multiple concurrent requests efficiently, and the Random Forest model’s predict_proba method is thread-safe, ensuring stable performance under moderate load. The frontend, built with React and Vite, achieves a Lighthouse performance score exceeding 95, thanks to efficient bundle splitting, lazy loading of admin routes, and optimized asset delivery. On the data layer, Supabase’s managed PostgreSQL scales horizontally, and Row Level Security policies are optimized with appropriate indexes on foreign keys and frequently queried columns such as analysis_logs.user_id and analysis_logs.created_at.

### 4.11. SiBot web application interface

**Fig 11:**
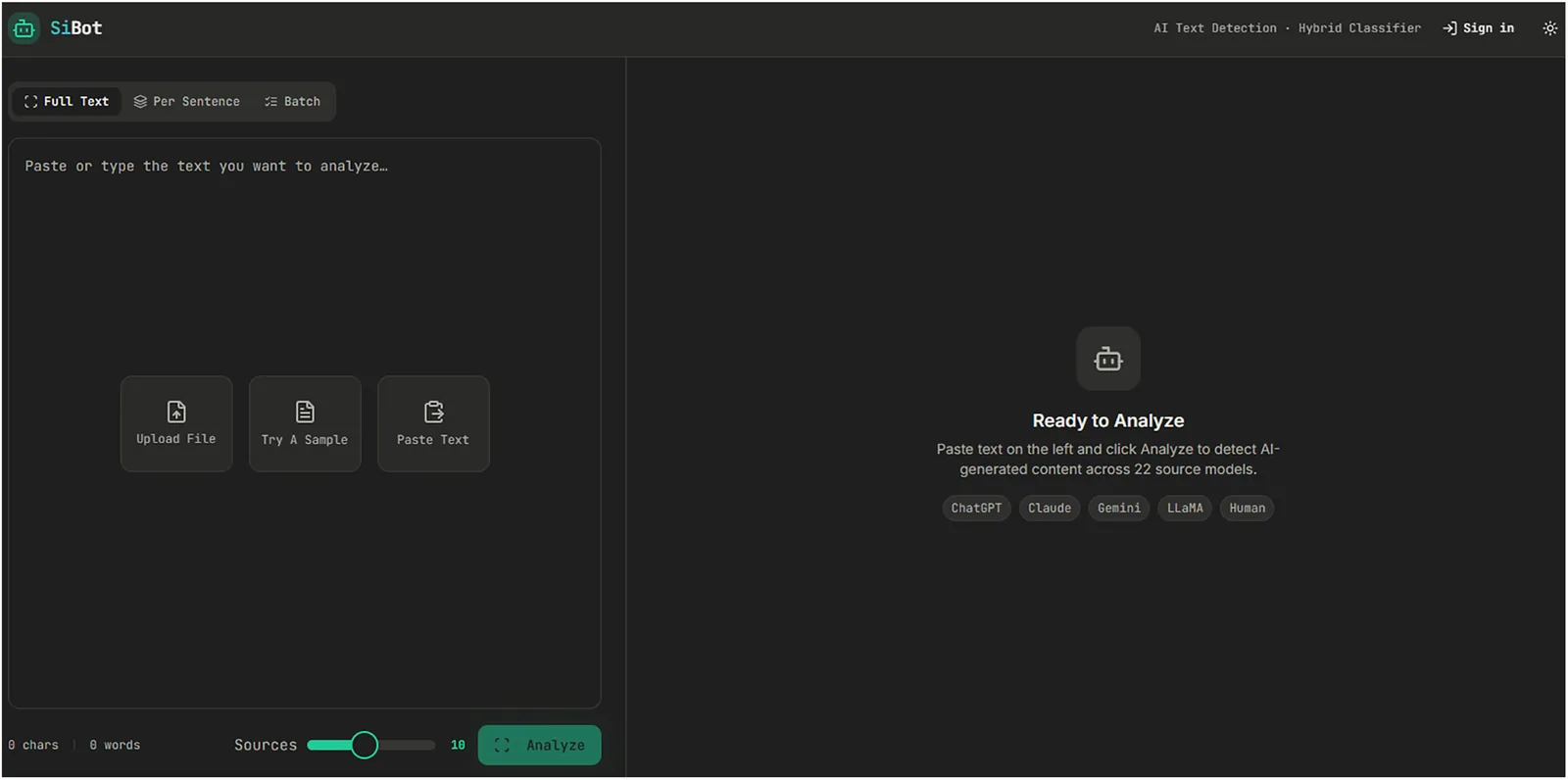
SiBot interface in its initial state.

**Fig 12:**
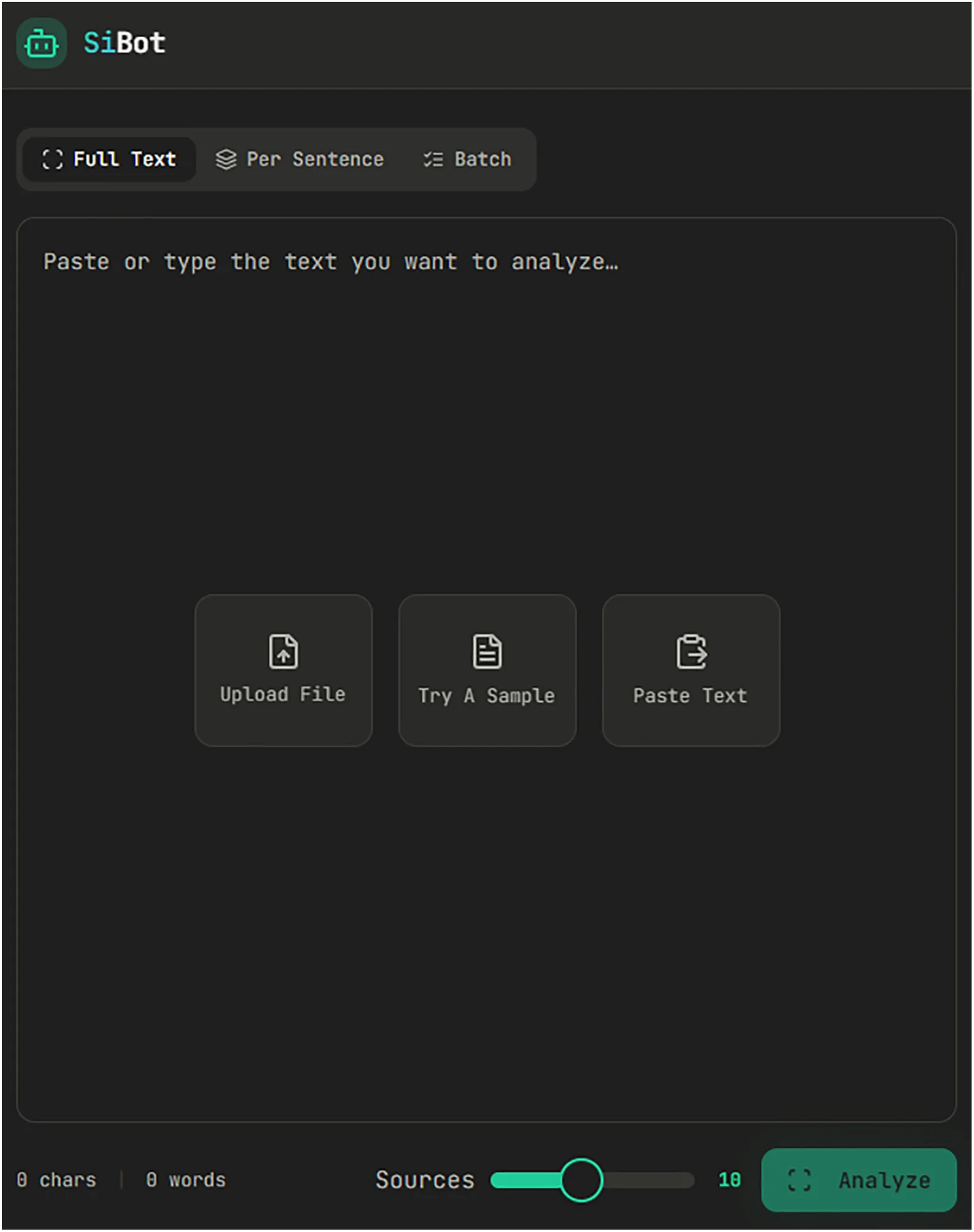
SiBot text input interface.

**Fig 13:**
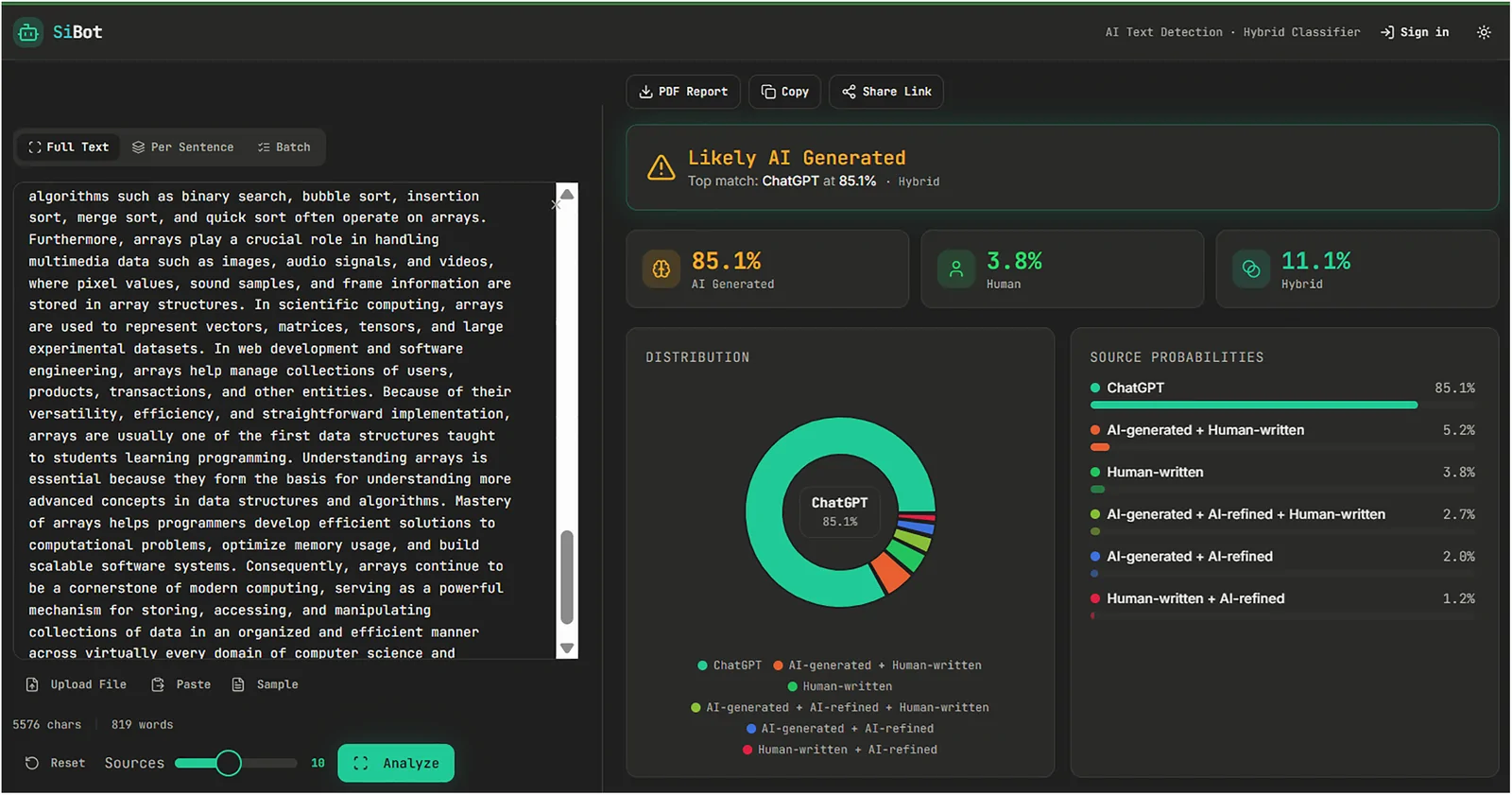
Output view of SiBot, showing verdict, component breakdown and ranked source probabilities.

**Fig 14:**
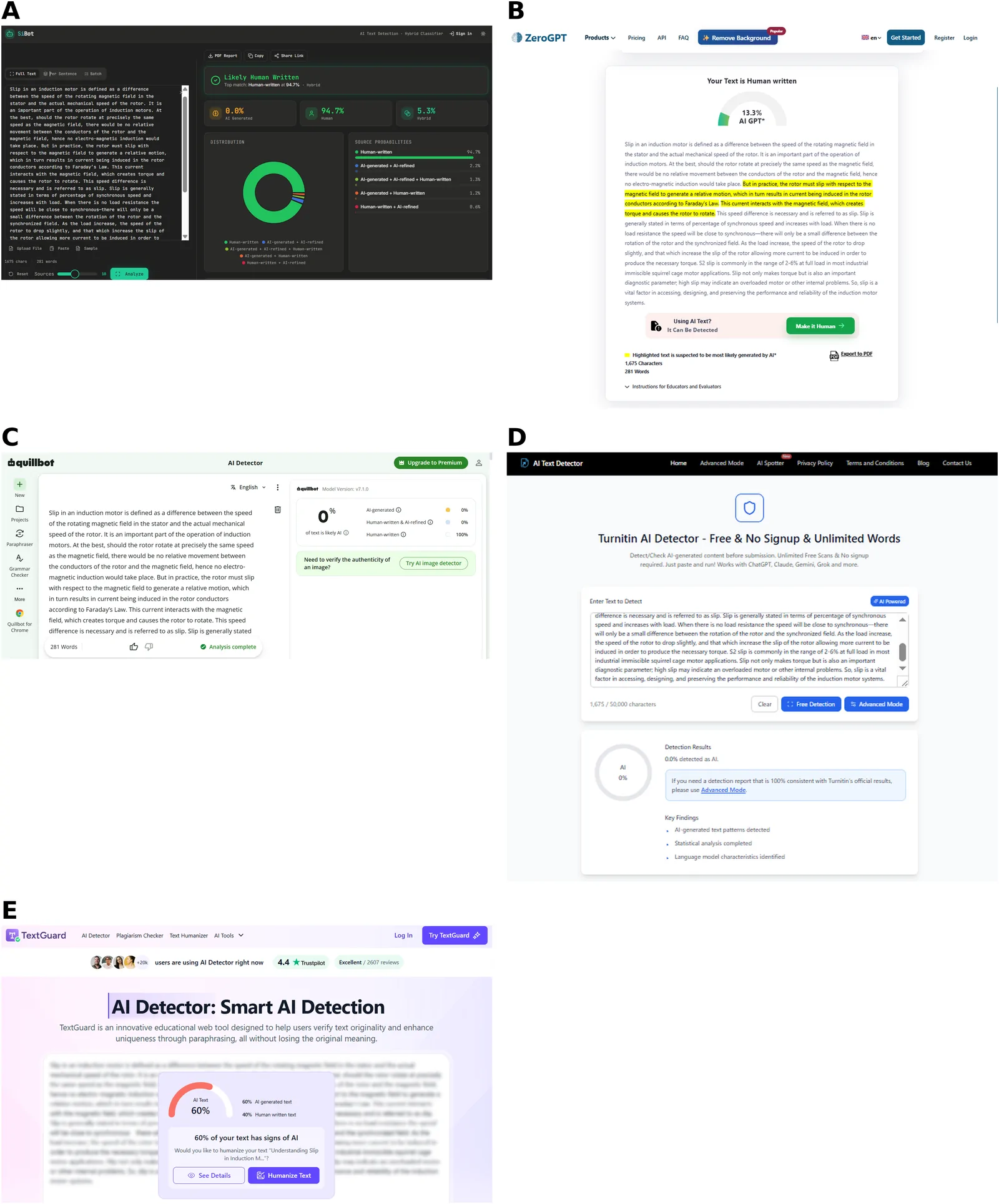
Interface outputs for the human-written induction-motor text. (A) SiBot; (B) ZeroGPT; (C) QuillBot; (D) Turnitin; (E) TextGuard.

To demonstrate the practical usability of the system, we present screenshots of the live SiBot interface. Figs 11–14 replace the interface screenshots of the earlier version and show the current deployment, which serves the 10-class hybrid model. Fig 11 presents the overall interface in its initial state. The left-hand panel hosts the text input area together with the full-text, per-sentence and batch analysis modes, while the right-hand panel prompts the user to begin analysis and indicates the supported provenance classes, with quick-reference chips for key providers alongside the human category.

Fig 12 provides a closer view of the text input interface, where the user selects the analysis mode and supplies text by typing or pasting directly, by uploading a file, or by loading a built-in sample, which keeps the submission workflow straightforward for non-technical users.

Fig 13 illustrates the output view once a text has been analysed. The interface reports an overall verdict banner with the top-matched source and its confidence, summary cards for the AI-generated, human-written and hybrid components, a distribution chart, and a ranked list of source probabilities. In the example shown, an AI-generated passage is attributed to ChatGPT at 85.1% confidence, accompanied by an 11.1% hybrid component and a marginal 3.8% human-written probability. This level of granularity goes far beyond binary AI-versus-human detectors. All interface components are powered by the backend API described in Section 3.2 and are designed to provide both interpretability and actionable insight for users ranging from individual writers to institutional administrators.

## 5. Discussion

This discussion synthesises the empirical findings around four themes: the nature of the stylometric evidence that makes attribution possible (5.1), why feature fusion outperforms either representation alone (5.2), what the system offers beyond binary detection (5.3 and 5.4), and the practical and ethical consequences of deploying attribution technology (5.5 to 5.7).

### 5.1. The “Semantic Relation” and AI Fingerprints

The results of this study provide strong evidence for the existence of distinct, model-specific fingerprints in AI-generated text. These fingerprints are not necessarily semantic but are deeply stylometric. The success of TF-IDF suggests that LLMs have subtle but measurable preferences for certain lexical items and syntactic constructions. For example, one model might consistently use more formal transition words (“furthermore,” “consequently”), while another might favor a more conversational tone with contractions and simpler sentence structures. The global SHAP analysis in Section 4.6 corroborates this directly: the features carrying the greatest attribution weight are function words and generic discourse markers, not topic-bearing nouns. Since the same prompts were issued to every generator, topical content is shared across classes by construction and cannot carry the signal, so the fact that the model’s most-weighted evidence is stylistic is a consequence of the experimental design rather than a coincidence.

### 5.2. Why feature fusion outperforms either representation alone

The ablation in Section 4.3 poses a question it does not by itself answer: why should combining two representations produce a 17.73-point gain over the better of them, when the standard expectation for ensembling correlated predictors is a gain of a few points at most? The explanation we advance is that the two representations fail on *different* instances rather than the same ones. TF-IDF resolves cases where a generator has a distinctive lexical habit but loses cases where two generators share vocabulary and differ only in how they organise it. DeBERTa resolves the latter but, because contextual encoders are trained to abstract away surface form in favour of meaning, systematically discards precisely the lexical idiosyncrasies that separate generators answering the same prompt. Their errors are therefore substantially decorrelated, and a classifier with access to both can recover instances that neither could recover alone.

Two pieces of evidence support this reading over the alternative explanation that the gain simply reflects a larger feature space. First, if dimensionality alone were responsible, the two alternative fusion configurations in Table 7 would also improve on their components, and they do not: SVM with BiLSTM and BERT reaches 67.82% and XGB with BiLSTM and DeBERTa 68.56%, both below the 74.65% of standalone DeBERTa. Adding features can evidently hurt. Second, the fusion succeeds specifically when the contextual branch is fine-tuned rather than frozen; the frozen-extractor results in Table 3 show transformer embeddings performing worse than TF-IDF alone, which is what one expects if the frozen representation has already discarded the attribution-relevant surface information before fusion can exploit it.

### 5.3. The hybrid classes

The inclusion of hybrid classes adds a layer of ecological validity to our study. The 10-class corpus separates four distinct collaboration workflows rather than treating mixed authorship as one undifferentiated category, and the per-class results in Table 8 show that these workflows are genuinely separable, at recalls between 91.8% and 92.4%. The pattern of separability is itself informative. Classes involving an AI refinement pass (7 and 8) reach AUC 0.99, indicating that AI refinement imposes a strong and consistent signature: it regularises sentence length and normalises discourse markers in a way that is highly detectable. Classes involving human editing of AI drafts (6 and 9) are markedly harder, at 0.80 and 0.83, which is the expected result: human editing is idiosyncratic and partial, so it degrades the AI signature without imposing a consistent replacement. This asymmetry has a practical implication for users. A SiBot verdict of “AI-refined” is considerably more reliable than a verdict distinguishing lightly human-edited AI text from the three-stage workflow, and the interface communicates the full probability distribution precisely so that this distinction remains visible rather than being collapsed into a single label [79].

### 5.4. Beyond binary detection: Quantitative comparison with commercial detectors

A key contribution of this work is a production-ready interface that fundamentally differs from existing tools like QuillBot and ZeroGPT [80,81]. SiBot’s interface does not merely provide a probability of a text being AI-generated. Instead, it performs a multi-class prediction, outputting the most probable source model and an explicit hybrid decomposition. The earlier version of this manuscript supported this claim with a single qualitative screenshot comparison, which the reviewers rightly identified as insufficiently rigorous. We therefore replace it with a controlled benchmark. Three versions of a response to one generating question, “What is slip in an induction motor?”, were prepared: a purely AI-generated response produced by Gemini, a purely human-written response, and a hybrid response in which human-written content was subsequently refined by AI. The provenance of each is known by construction. All three were submitted to five systems: QuillBot, Turnitin, ZeroGPT, TextGuard and SiBot. For every tool, confidence scores were recorded across four analysis dimensions: probability that the text is AI-generated, identification of the specific generating model, probability that the text is human-written, and probability of mixed origin. Table 12 gives the benchmark material and Tables 13–15 the results, in which an “x” indicates that the tool does not offer that analysis dimension at all.

**Table 12:** Benchmark generating question and the three response variants.

Question	Response	Origin
What is slip in an induction motor?	Slip is a fundamental concept in the operation of an induction motor, representing the relative speed difference between the magnetic field and the rotor itself. The synchronous speed (Ns) is the rotational speed of the magnetic field created by the stator windings, while the rotor speed (Nr) is the actual mechanical speed at which the rotor turns. For an induction motor to produce torque, the rotating magnetic field must continuously cut the rotor conductors to induce voltage and current; therefore, the rotor speed must always be less than the synchronous speed, making the motor asynchronous. Slip is mathematically defined as the ratio of the difference between the synchronous speed and the rotor speed to the synchronous speed, expressed either as a fraction or a percentage. The formula for slip (s) is: s = (Ns − Nr)/ Ns. At standstill, Nr = 0, so the slip s = 1 (or 100%), and under light load or no-load conditions, the rotor speed is close to synchronous speed, making the slip very small (typically 1–5%). Slip is crucial because the frequency of the induced voltage in the rotor is directly proportional to it, and the developed torque is also a function of the slip.	AI generated (Gemini)
	Slip in an induction motor is defined as a difference between the speed of the rotating magnetic field in the stator and the actual mechanical speed of the rotor. It is an important part of the operation of induction motors. At the best, should the rotor rotate at precisely the same speed as the magnetic field, there would be no relative movement between the conductors of the rotor and the magnetic field, hence no electro-magnetic induction would take place. But in practice, the rotor must slip with respect to the magnetic field to generate a relative motion, which in turn results in current being induced in the rotor conductors according to Faraday’s Law. This current interacts with the magnetic field, which creates torque and causes the rotor to rotate. This speed difference is necessary and is referred to as slip. Slip is generally stated in terms of percentage of synchronous speed and increases with load. When there is no load resistance the speed will be close to synchronous. As the load increases, the speed of the rotor drops slightly, and that increases the slip of the rotor allowing more current to be induced in order to produce the necessary torque. Slip is commonly in the range of 2–6% at full load in most industrial squirrel cage motor applications. Slip not only makes torque but is also an important diagnostic parameter; high slip may indicate an overloaded motor or other internal problems.	Human written
	The rotor moves more slowly than the magnetic field in the stator spins faster. We call this slip. This is a very important part of how induction motors work. What if the rotor turned at the same speed as the magnetic field? The conductors of the rotor and the magnetic field would stay still. To put it another way, there would be no electromagnetic generation. In real life, though, the rotor needs to move away from the magnetic field to do that. Based on Faraday’s Law, this makes electricity flow through the rotor’s lines. There is force when this current hits the magnetic field. This force turns the rotor. We need this difference in speed, which is called slip. The amount of slip is often shown as a share of the steady speed. Slip gets worse as the load goes up. When there is no load, there will not be much to slow it down. Because of the extra weight, the rotor moves more slowly. The rotor slips more, which lets more current flow to make the power they need. Most of the time, slip in commercial squirrel cage motors is between 2% and 6% at full load. Slip can be used to make force or figure out what’s wrong.	Hybrid (human written, AI refined)

**Table 13:** Detection confidence comparison for the AI-generated response (true origin: Gemini).

Dimension	QuillBot	Turnitin	ZeroGPT	TextGuard	SiBot (proposed)
AI (%)	100	49	100	65	**96.7**
AI source identified	x	x	x	x	**Gemini (correct)**
Human (%)	0	x	x	35	1.5
Hybrid (%)	0	x	x	x	1.8

**Table 14:** Detection confidence comparison for the human-written response (true origin: human).

Dimension	QuillBot	Turnitin	ZeroGPT	TextGuard	SiBot (proposed)
AI (%)	0	0	19.2	60	**0**
AI source identified	x	x	x	x	**None (correct)**
Human (%)	100	x	x	x	**94.7**
Hybrid (%)	0	x	x	x	5.3

**Table 15:** Detection confidence comparison for the hybrid response (true origin: human written, AI refined).

Dimension	QuillBot	Turnitin	ZeroGPT	TextGuard	SiBot (proposed)
AI (%)	0	0	5.5	76	0.9
Human (%)	100	x	x	x	1.4
Hybrid (%)	0	x	x	x	**97.7**
Hybrid source identified	x	x	x	x	**Human-written + AI-refined (correct)**

The results warrant close reading. On the AI-generated response (Table 13), QuillBot and ZeroGPT both returned 100% AI confidence and Turnitin 49%, while TextGuard split its judgment between 65% AI and 35% human; none could attribute the text to its generating model, as the “x” entries record. SiBot assigned 96.7% AI confidence and was the only system to identify Gemini correctly. On the human-written response (Table 14), QuillBot and Turnitin correctly reported 0% AI, ZeroGPT produced a mild false-positive signal of 19.2%, and TextGuard failed outright, labelling a genuinely human-written passage as 60% AI, a false positive that in an academic-integrity setting could have serious consequences for a student. SiBot returned 94.7% human confidence with 0% AI and a 5.3% residual hybrid component.

The hybrid response (Table 15) is the most discriminative case and the one that most clearly exposes the limitation of the binary paradigm. QuillBot classified the text as 100% human, Turnitin reported 0% AI, and ZeroGPT reported only 5.5% AI: all three effectively overlooked the AI involvement entirely. TextGuard overcorrected in the opposite direction, labelling the same text 76% AI. SiBot was the only system to recognise the mixed provenance, assigning 97.7% hybrid confidence with marginal AI (0.9%) and human (1.4%) probabilities and correctly characterising the text as human-written and AI-refined. Two conclusions follow. The existing detectors do not merely underperform; they produce mutually contradictory verdicts on identical inputs, spanning 0% to 76% AI on the same passage, which means that a user consulting more than one of them has no principled way to reconcile them. And they are structurally incapable of source attribution and hybrid detection, not merely inaccurate at it: the “x” entries that dominate their columns are missing capabilities, not failed measurements.

Figs 14A–E shows the corresponding interface outputs for the human-written sample, which make the contrast directly visible. It should be noted that this benchmark, while quantitative and controlled for provenance, remains limited in scale: it evaluates three carefully constructed texts on a single topic. A broader evaluation across a standardised public benchmark is identified in Section 6 as necessary further work, and the conclusions above should be read as establishing a capability gap rather than a precise accuracy ranking.

### 5.5. Lessons learned in production deployment

Deploying a research model as a public web application presented several challenges and yielded valuable insights. Model serialization and size were primary concerns: large artefacts required Git LFS for version control on Hugging Face Spaces, and compression proved essential to reduce storage and loading time. Ensuring environment parity between the training environment and the production Docker container was critical to avoid silent prediction errors; mismatches in scikit-learn versions, for instance, could cause inconsistent feature transformations. Rate limiting and abuse prevention became necessary to manage costs on the free tier; implementing per-user rate limits based on roles stored in Supabase effectively controlled API usage. Finally, observability was enhanced by integrating structured logging and an audit trail, which proved crucial for debugging, security compliance, and understanding user behavior. Deploying the hybrid model introduced one further constraint absent from the single-branch pipeline: because the two branches must be applied in exactly the order and configuration used during training, the vectorizer, the tokenizer configuration and the encoder checkpoint are versioned together as a single artefact, and the API refuses to start if any component hash does not match the manifest. Silent branch mismatch would produce plausible but meaningless probability distributions, which is a more dangerous failure mode than an outright crash.

### 5.6. Bias, ethical considerations and potential for misuse

A system that attributes text to a source is a system that can be wrong about a person, and the consequences of being wrong are not symmetric. We therefore set out the risks explicitly rather than leaving them implicit.

**Bias.** Three sources of bias are relevant. The corpus is monolingual English and was authored predominantly in a single institutional and regional context; prior work has established that AI-text detectors systematically misclassify writing by non-native English speakers as machine-generated [23], and nothing in the present design immunizes SiBot against that failure mode. We have not evaluated performance disaggregated by author background, and we do not claim fairness across such groups. The human-written class is drawn from academic and student writing, so the model’s implicit notion of “human” is a formal register; informal, dialectal or creative human writing lies outside the distribution it has seen. And the prompt bank, while spanning six domains, encodes the authors’ choices about what constitutes a representative question.

**Reliability and appropriate use.** At 92.38% accuracy, roughly one verdict in thirteen is wrong. In an academic-integrity context, that rate is unacceptable as a basis for a disciplinary finding, and we state plainly that SiBot’s output should be treated as one piece of evidence prompting further inquiry, never as proof of misconduct. The interface is deliberately designed to support this: it returns a full probability distribution rather than a verdict, and the sentence-level view localizes the evidence, so that a reviewer can inspect the basis of a judgment rather than defer to it. The SHAP and LIME facilities exist for the same reason.

**Misuse.** Attribution technology can be turned to purposes its authors did not intend. Source attribution could be used to identify which model an author or organization relies on, which is commercially or politically sensitive information; it could support surveillance of writing practices in workplaces or classrooms in ways that are disproportionate; and a public attribution system provides an oracle against which adversaries can iterate to produce text that evades it, as the adversarial robustness results in RAID [37] demonstrate for detectors generally. Rate limiting and audit logging mitigate the last of these only partially. We note also that a system distinguishing AI-refined from human-written text could be used to penalise legitimate assistive use of AI by writers with disabilities or by non-native speakers, which would invert the intended purpose entirely.

**Temporal validity.** Large language models are updated frequently, and a stylistic fingerprint captured at one point in time may not survive a model revision. Any deployed attribution system therefore has a shelf life, and treating its verdicts as stable across model generations is unsafe. The SiBot deployment records the corpus construction date alongside its predictions for this reason, and periodic re-training against current model versions is a maintenance requirement rather than an optional enhancement.

### 5.7. Limitations

The principal limitations are as follows. The corpus is monolingual and drawn from controlled prompt settings, so it may not capture the full variability of real-world content generation. The set of generators is fixed at a point in time, and performance against models released subsequently is unknown. The framework has not been validated on external public benchmarks such as RAID or OpenTuringBench, which is necessary before the accuracy figures can be compared directly with published results from other groups. Adversarial robustness has not been evaluated: we have not tested how the model behaves under deliberate paraphrasing attacks, which the recent literature identifies as the dominant failure mode for detectors. The explainability analysis relies on post-hoc methods that approximate rather than reveal the model’s internal decision process. And the deployed system supports ten classes rather than the full twenty-two, which is a deliberate accuracy-driven choice but nonetheless a restriction on coverage.

## 6. Conclusion and future work

This paper presented SiBot, a complete research-to-production framework for fine-grained attribution of AI-generated text. We introduced a novel, large-scale dataset of 66,000 samples spanning 22 sources, together with a 30,000-sample, 10-class corpus in which four human-AI collaboration workflows are modelled explicitly. Through extensive experimentation, we demonstrated that a combination of TF-IDF feature extraction and a Random Forest classifier achieves 68.2% accuracy across 22 classes, outperforming models built on frozen deep learning embeddings, and that a hybrid architecture fusing TF-IDF lexical features with fine-tuned DeBERTa contextual features before Random Forest classification achieves 92.38% accuracy on the 10-class task, 17.73 points above the strongest fine-tuned transformer and 34.30 points above the lexical branch alone, with non-overlapping confidence intervals. The ablation establishes that this performance derives from the interaction of the two branches rather than from either individually, and the SHAP and LIME analyses show that the operative evidence is stylistic rather than topical.

Because the hybrid model is both substantially more accurate and interpretable, it is the model integrated into the live SiBot service. Against four commercial detectors on a controlled provenance benchmark, it was the only system able to name the generating model of AI text and the only one able to recognise hybrid human-AI provenance, while the commercial tools returned verdicts spanning 0% to 76% AI on an identical input.

Future work will explore several avenues. Extending the production deployment from 10 to all 22 classes is the primary objective: the 22-class model is reported here as a research benchmark, and applying the hybrid fusion architecture to the 22-class corpus, rather than the single-representation pipeline evaluated in Section 4.1, is the most promising route to closing the accuracy gap to a level that justifies public deployment at that coverage. End-to-end fine-tuning of additional LLMs directly on the attribution task could further improve performance. Cross-domain and cross-topic evaluation will test generalizability on out-of-domain data, and validation against public benchmarks such as RAID [37] and OpenTuringBench [42] will place these results on a common footing with the wider literature. Temporal analysis is also important, as LLMs undergo frequent updates; investigating the stability of stylistic fingerprints over time will be crucial for real-world deployment. Adversarial robustness studies will examine how easily these attribution methods can be fooled by paraphrasing, guiding the development of more resilient models. A disaggregated fairness evaluation across author backgrounds, particularly for non-native English writers, is a prerequisite for responsible use in academic-integrity settings. Finally, we plan to continuously expand model coverage by adding new AI sources and to extend support to multilingual text.
