# Supplementary material for: SiBot: A hybrid framework for fine-grained semantic attribution of AI-generated text and its production deployment
Source: PLoS One. 2026 Sep 8;21(9):e0357405. doi: 10.1371/journal.pone.0357405 (PMC13552777; doi:10.1371/journal.pone.0357405)
Supplement: Supplementary file 1 [file pone.0357405.s001.docx]

**Review Report**

The manuscript presents **SiBot**, a production-ready framework for fine-grained attribution of AI-generated text across 22 language models by integrating TF-IDF feature extraction with Random Forest classification and a publicly accessible web platform. The study addresses an important and timely challenge in AI text forensics by moving beyond conventional binary AI detection toward identifying the likely source model of generated content. The proposed dataset, large-scale experimental evaluation, and end-to-end deployment demonstrate considerable practical effort and provide a useful contribution to the emerging field of AI-generated text attribution. The manuscript is generally well organized and technically sound, with a clear description of the system architecture and implementation. However, several aspects require further clarification and strengthening before publication.

**1.** The novelty of the proposed approach should be articulated more explicitly in comparison with the most recent studies on multi-class AI source attribution, as the current discussion does not sufficiently distinguish the methodological contribution from existing work.

**2.** The manuscript would benefit from a more comprehensive description of the dataset generation process, including prompt selection strategy, topic diversity, quality control, and measures taken to minimize prompt-induced bias across language models.

**3.** Although the reported accuracy is encouraging for a 22-class problem, additional statistical analyses such as confidence intervals, significance testing, or repeated cross-validation would strengthen the credibility of the reported performance.

**4.** The experimental evaluation should include stronger benchmarking against recent transformer-based fine-tuned attribution models rather than relying primarily on feature extraction approaches, allowing readers to better assess the competitiveness of the proposed method.

**5.** A detailed confusion matrix and per-class precision, recall, and F1-scores should be presented to reveal which language models are frequently confused and to better understand the limitations of the classifier.

**6.** The discussion of generalization remains limited, and the authors should evaluate the model using unseen prompts, cross-domain datasets, or newly released language models to demonstrate robustness beyond the constructed dataset.

**7.** The manuscript would benefit from a more critical discussion of potential biases, ethical considerations, and possible misuse of AI attribution systems, particularly considering the evolving nature of large language models and their frequent updates.

**8.** The comparison with commercial tools such as ZeroGPT and QuillBot is informative but lacks methodological rigor because only a limited qualitative comparison is presented; a broader quantitative evaluation using standardized benchmark datasets would substantially strengthen the claims.

**9.** Greater emphasis should be placed on computational efficiency, training time, memory consumption, and scalability compared with competing methods, particularly since production deployment is one of the central contributions of the work.

**10.** The manuscript would benefit from careful language editing to improve readability, reduce repetitive descriptions of the system architecture, and ensure consistency in terminology throughout the paper.

Overall, the work addresses a highly relevant research problem and demonstrates practical significance, but addressing the above comments would considerably improve its scientific rigor, reproducibility, and impact, making it more suitable for publication.
