# Supplementary material for: SiBot: A hybrid framework for fine-grained semantic attribution of AI-generated text and its production deployment
Source: PLoS One. 2026 Sep 8;21(9):e0357405. doi: 10.1371/journal.pone.0357405 (PMC13552777; doi:10.1371/journal.pone.0357405)
Supplement: Supplementary file 2 [file pone.0357405.s002.docx]

**Response to Reviewers**

**Overall Response:** We are grateful to the Editor and to all three reviewers for the time and care they have devoted to this manuscript. The comments were detailed, constructive and, in several instances, identified weaknesses that we had not adequately recognized ourselves. Every comment has resulted in a substantive change to the paper. All modified and newly added text is highlighted in yellow in the revised manuscript so that the changes can be located quickly.

Three changes affect the manuscript globally, and we describe them once here rather than repeating them under each comment. First, the deployed SiBot system now serves the 10-class hybrid model (TF-IDF + DeBERTa + Random Forest, 92.38% accuracy) rather than the 22-class model. Both sets of results are reported in full, the reasoning for the deployment choice is set out in Section 3.1.8, and extending deployment to all 22 classes is stated as the leading item of future work. Second, a claim made in the original manuscript, that lightweight feature-based approaches "remain competitive" with deep learning, proved not to be supported by our own data once we ran the additional experiments the reviewers requested. We have withdrawn the claim rather than defend it, and replaced it in Section 4.8 with the narrower statements the evidence does support. Third, the dataset underlying the study has been publicly released as the TXD-22 benchmark at https://doi.org/10.17632/prcjcggtjf.1, and the model controller source code has been made openly available, so that the results reported here are reproducible in full. Fourth, the manuscript has been thoroughly edited for language, repetition and terminological consistency throughout.

**Reviewer 1**

We thank Reviewer 1 for the encouraging assessment of the writing and technical tone, and for a set of comments that were unusually specific and therefore unusually actionable. The manuscript is considerably stronger for them.

**Comment 1.** *"The experimental section is currently limited, with only a single results table presented. Additional experiments and analyses are needed to adequately demonstrate the effectiveness, robustness, and generalizability of the proposed approach."*

**Response.** Thank you for this comment; it was the single most consequential criticism in the reviews and it prompted a substantial expansion of the work. The experimental section has grown from one table to sixteen tables and fifteen figures. Specifically, we have added Table 4 (five classical machine-learning models), Table 5 (five deep-learning architectures), Table 6 (five fine-tuned transformer models), Table 7 with Figure 3 (a component-wise ablation), Table 8 with Figures 4 to 7 (per-class metrics, confusion matrix and ROC analysis), Table 9 (confidence intervals), Table 10 with Figure 9 (computational efficiency), Table 11 with Figure 10 (comparison against eight published studies), and Tables 12 to 15 (a controlled quantitative benchmark against four commercial detectors). Robustness is addressed through prompt-aware ten-fold cross-validation and a dedicated generalization analysis in Section 4.7. These additions appear in Sections 4.2 to 4.9.

**Comment 2.** *"The paper lacks sufficient details regarding the data generation and curation process… The authors should provide examples of prompts and describe the methodology used in prompt engineering."*

**Response.** Thank you for raising this; the original description was indeed too thin to allow the corpus construction to be assessed or reproduced. Section 3.1.1 now documents the full protocol. The prompt bank comprises 3,000 unique prompts spanning thirty academic domains, each with eight to ten sub-domains, crossed with five task types, and the same 3,000 prompts were put verbatim to every source. The authoring procedure is described in four steps: drafting seed prompts per cell, screening for near-duplication using cosine similarity over TF-IDF vectors with a rejection threshold of 0.85, manual review to remove ambiguity and any vendor-specific or product-specific wording that might bias a particular model family, and finally issuing the frozen prompt verbatim to every source. We also explain why identical prompting matters for the validity of the attribution claim. Table 1 now gives eight verbatim example prompts drawn from across the design.

**Comment 3.** *"The paper states that 6,600 samples were used; however, no information is provided regarding data availability. If the dataset cannot be publicly released, representative examples should be included in the manuscript."*

**Response.** Thank you for this comment, which prompted us to go further than the alternative you offered. Rather than substituting representative examples for release, we have released the dataset itself. The full corpus has been deposited in the Mendeley Data repository under the name TXD-22 and is openly available at https://doi.org/10.17632/prcjcggtjf.1. It is cited in the manuscript as: Iqbal, M. S., & Kashem, M. A. (2026). *TXD-22: A large-scale benchmark dataset for multi-class AI-generated and mixed-source text identification* (Version 1) [Data set]. Mendeley Data. https://doi.org/10.17632/prcjcggtjf.1. The citation appears as reference [81], as a footnote in Section 3.1.1, in the Data Availability Statement, and in the contributions list in Section 1.1, since public release is itself a contribution of the work. Every result reported in Section 4 is therefore now directly reproducible. We have nonetheless retained the representative examples you suggested: Table 2 presents one sample per class for all ten classes, showing prompt, response and label, with each example deliberately drawn from a different subject domain so that the topical breadth of the corpus is visible at a glance, and Table 12 reproduces the complete benchmark texts verbatim, so that a reader can assess the structure of the corpus without downloading it. We should note that the sample count in the original manuscript was misstated; the corpora comprise 66,000 and 30,000 samples respectively, and this has been corrected throughout.

**Comment 4.** *"The quality and resolution of Figures 1 and 2 should be improved… and if created using AI, please disclose that in the acknowledgments for AI usage."*

**Response.** Thank you for both points. Figures 1 and 2 (the pipeline and architecture diagrams) have been completely redrawn at 600 dpi with substantially enlarged typography, and the architecture diagram now additionally carries a numbered request lifecycle so that the data flow can be followed without reference to the body text. On the disclosure point, we have added a dedicated section, "Acknowledgements and Disclosure of AI Usage", stating the provenance of every figure individually: which were produced with AI-assisted drawing tools and subsequently verified by the authors, which were generated programmatically from the experimental results, which are direct outputs of our own experimental code, and which are unmodified screenshots. The statement also discloses that generative AI systems were used to produce the research corpus itself, which is the subject matter of the study, and for language editing, and confirms that all technical content and conclusions are the authors' own.

**Comment 5.** *"The literature review should be strengthened by incorporating recent studies published in 2024, 2025, and 2026."*

**Response.** Thank you for this; the review was indeed weighted toward work published before 2024. We have added a new Section 2.5 devoted to recent advances, organised around the three developments most relevant to our design decisions. The first is robustness benchmarking, covering RAID (ACL 2024) and MGTBench (CCS 2024), which motivated our prompt-aware splitting protocol and the candid generalisation analysis in Section 4.7. The second is efficient zero-shot detection, covering Fast-DetectGPT (ICLR 2024) and Binoculars (ICML 2024), which we discuss as strong binary detectors that are structurally unable to perform attribution. The third is the consolidation of attribution as a distinct task, covering the SIGKDD Explorations survey (2025), OpenTuringBench (EMNLP 2025), LLM code stylometry (2025), LLM-as-a-Coauthor (2024), EditLens (2025) and recent linguistic-feature work. Fifteen references from 2024 to 2026 have been added and integrated into the argument rather than merely appended.

**Comment 6.** *"Hugging Face repositories, or FastAPI web links should be provided as footnotes."*

**Response.** Thank you for this suggestion, which has made the paper considerably more useful to anyone wishing to build on it. Four footnotes have been added. The first, in the Introduction, gives the live application at https://www.sibot.us/. The second gives the Hugging Face Spaces inference service together with the model controller repository. The third, in Section 3.2.3, gives the interactive FastAPI/OpenAPI documentation served at the /docs path of the hosted service, where every endpoint described in that section can be exercised directly. The fourth, in Section 3.1.1, gives the dataset. The two code and data resources are cited in full as follows. The source repository: Iqbal, M. S., & Kashem, M. A. (2026). *SiBot model controller* [Computer software]. GitHub. Retrieved July 27, 2026, from https://github.com/XDR-SAM/SIBOT_Model_Controller.git, listed as reference [82], which contains the model controller source code, the deployment configuration and the supplementary material. The dataset: Iqbal, M. S., & Kashem, M. A. (2026). *TXD-22: A large-scale benchmark dataset for multi-class AI-generated and mixed-source text identification* (Version 1) [Data set]. Mendeley Data. https://doi.org/10.17632/prcjcggtjf.1, listed as reference [81]. Both are also given in the Data Availability Statement so that they are visible to readers who consult that section rather than the footnotes.

**Comment 7.** *"An ablation study is recommended… Moreover, the proposed method should be compared against other multiclass detection/classification approaches."*

**Response.** Thank you for this recommendation, which materially improved the evidential basis of the paper. Table 7 and Figure 3 now present a component-wise ablation on identical data and splits, comparing the proposed configuration against its lexical branch alone (58.08%), its contextual branch alone (74.65%), and two alternative fusion designs (67.82% and 68.56%). The proposed configuration reaches 92.38%. We draw particular attention to the fact that both alternative fusion designs fall *below* the standalone transformer baseline, which demonstrates that fusion is not automatically beneficial and that the specific combination of branches and classifier is what carries the result. On the second part of the comment, Table 11 and Figure 10 compare the method against eight published detection and classification studies, with the commensurability caveats stated explicitly in the accompanying text.

**Comment 8.** *"The manuscript claims that lightweight feature-based approaches remain competitive with deep learning methods for production deployment. This claim should be supported with experimental evidence… authors should specifically demonstrate that reductions in computational complexity, model size, or deployment cost do not lead to significant degradation in predictive performance."*

**Response.** Thank you for pressing on this. We tested the claim as you asked, and we must report that it did not survive the test: on the 10-class task, TF-IDF with Random Forest attains 58.08% against 74.65% for a fine-tuned transformer, which is not competitive by any reasonable reading. We have therefore withdrawn the claim from the abstract, the introduction and the conclusion rather than attempt to defend it. In its place, Section 4.8 and Table 10 report accuracy against artefact size, inference latency, training time and hardware requirement, and state the two narrower claims that the evidence does support: that on the 22-class task sparse lexical features outperform *frozen* transformer embeddings at roughly a third of the artefact size, which is a genuine result about frozen-extractor pipelines specifically; and that the deployed hybrid attains the highest accuracy of any configuration evaluated while remaining servable on CPU-only infrastructure at roughly half the footprint of a fine-tuned DeBERTa. The efficiency argument thus survives, but as a property of the hybrid rather than of the lightweight branch alone. Figure 9 plots this trade-off. We are grateful for a comment that led us to correct a genuine overstatement.

**Comment 9.** *"The paper mentions the use of 'diverse' data; however, this term is not clearly defined."*

**Response.** Thank you; the term was indeed used loosely. Section 3.1.1 now defines diversity along three explicit axes. Topical diversity means coverage of thirty academic domains, each broken down into eight to ten sub-domains, spanning engineering, the natural sciences, the social sciences, law, medicine, the humanities and the creative and media disciplines. The full distribution is now given as Table 1. Task diversity means coverage of five task types within each domain: factual explanation, comparative analysis, procedural description, opinion or argumentative writing, and summarization. Structural diversity means deliberate variation in prompt length and specificity, from single-clause questions of under ten words to multi-constraint instructions exceeding fifty words. Table 1 sets out the full domain and sub-domain distribution, and Table 2 illustrates the same breadth by drawing its ten class examples from ten different domains of that table rather than from a single field. We have also made explicit that the same 3,000 prompts were put verbatim to every generative source, so that the classes differ only in what produced the response and not in what was asked.

**Reviewer 2**

We thank Reviewer 2 for a set of comments that addressed both the substance of the work and its presentation. The comments on the abstract, on the justification of our methodological choices, and on the treatment of deep learning were particularly valuable, and each has led to significant revision.

**Comment 1.** *"The abstract is not expressing the novelty of the proposed approach. The whole abstract is not impressive and needs to be rewritten. Should focus on what is problem, why it is important to be solved. How it is solved and what are findings."*

**Response.** Thank you for this direct assessment, which we accept. The abstract has been rewritten from scratch and now follows precisely the structure you specify. It opens by stating the problem, namely that deployed detectors answer only a binary question. It then establishes why this matters, namely that forensic and academic-integrity settings require knowing which model produced a text and whether a human was involved, neither of which a single probability can express. It describes the solution: two purpose-built corpora constructed from a controlled prompt bank, and a hybrid architecture fusing sparse lexical and contextual representations. And it closes with quantified findings, including the 92.38% result, the 17.73-point margin over the strongest transformer baseline, the ablation outcome, and the comparison against four commercial detectors.

**Comment 2.** *"Introduction section missing the novelty of the proposed approach; hence, the author is suggested to revise the introduction section."*

**Response.** Thank you. The introduction has been revised in two respects. A new paragraph states the specific problem precisely and identifies the three obstacles that have prevented the reformulation from being realized. More importantly, a new Section 1.1, "Novelty and Contributions", states three specific and testable novelty claims: that lexical and contextual evidence are complementary rather than redundant; that human-AI collaboration is separable into distinguishable workflow classes rather than a single hybrid bucket; and that attribution-grade granularity is achievable within production constraints. Each claim is cross-referenced to the experiment that tests it, so the novelty is stated as something falsifiable rather than asserted.

**Comment 3.** *"There should be some lines of text between the main and sub heading. This rule should be followed in whole paper."*

**Response.** Thank you for noting this. Lead-in paragraphs have been added or extended beneath Sections 2, 3, 3.1, 3.2, 4 and 5, so that no heading anywhere in the manuscript is immediately followed by a sub-heading. Each lead-in also summarizes what the subsections that follow will cover, which we hope improves navigability as well as conforming to the convention.

**Comment 4.** *"Figure 2 and 3 needs further illustration whereas the tables need consistent font. This rule should be followed in whole paper."*

**Response.** Thank you for both observations. The architecture figures have been redrawn with considerably fuller annotation, including explicit tier labelling, the technology stack at each layer, and a numbered five-step request lifecycle traced through the diagram; a new Figure 2 illustrates the hybrid model architecture itself, which was not depicted at all in the original. On the tables, all sixteen now use a single consistent font and size throughout (Times New Roman, 9.5 pt), with uniform header shading, uniform cell padding and uniform border weights.

**Comment 5.** *"Results section needs revision which demonstrates the training as well the testing results… the impacts of each consider parameter in a detailed manner… Author is requested to add an ablation study."*

**Response.** Thank you for this comment. Section 3.1.7 now documents the training and validation protocol in full: prompt-aware ten-fold cross-validation, the 8:2 split for the 22-class study, TF-IDF vocabulary sizes of 10,000 and 5,000 for the two corpora, sequence padding at 200 tokens, and 100 estimators for the Random Forest. Sections 4.2 to 4.5 report results across every model family, with per-class breakdowns in Table 8, confusion matrix and ROC analysis in Figures 5 and 7, and confidence intervals in Table 9. The ablation you request is provided in Table 7 and Figure 3, isolating each branch and testing two alternative fusion configurations.

**Comment 6.** *"Reference section should comprise of recent years' contributions."*

**Response.** Thank you. Fifteen references from 2024 to 2026 have been added, drawn from peer-reviewed venues including ACL, ICML, ICLR, EMNLP, ACM CCS and SIGKDD Explorations, together with arXiv preprints of record. These are not appended for form; they are integrated into the new Section 2.5 and into the discussion of robustness in Sections 4.7 and 5.6, where they inform specific design decisions and specific limitations we now acknowledge.

**Comment 7.** *"Author should explain the significance of utilizing TF-IDF in feature extraction because more significant techniques are available now which can improve the performance of their proposed approach."*

**Response.** Thank you for this challenge, which was entirely fair as the original manuscript offered no justification. Section 3.1.3 now argues the case task-specifically rather than generically. Because identical prompts are issued to every generator, the semantic content of their responses converges by construction, and the discriminative signal survives almost entirely in how the content is expressed: function-word frequencies, discourse markers, punctuation habits and characteristic bigrams. These are precisely the quantities a sparse n-gram representation encodes explicitly and that a dense contextual encoder is trained to abstract away. Table 3 supports this: TF-IDF outperforms both ALBERT and RoBERTa embeddings by 13 to 15 accuracy points on identical data. We also note that TF-IDF is interpretable at the token level, which is what makes the SHAP and LIME analyses possible. Most importantly, we take your underlying point: the revised deployed model does *not* rely on TF-IDF alone, but fuses it with a fine-tuned DeBERTa branch, which is worth a further 34 accuracy points.

**Comment 8.** *"Provide the logical evidence of utilizing Random Forest and Decision Tree for classification purpose and neglecting the deep learning algorithms. Consideration of the deep learning models may improve the performance of their proposed approach."*

**Response.** Thank you for this comment, which we regard as the most important in Reviewer 2's set, and your expectation proved correct. Deep learning is no longer neglected: Tables 5 and 6 now report five deep architectures and five fine-tuned transformers trained on identical data under identical protocols. The outcome confirms your prediction: fine-tuned transformers do outperform every classical model individually, with DeBERTa reaching 74.65% against 72.66% for the best classical model. Acting on this, we have incorporated a fine-tuned DeBERTa branch directly into the proposed architecture, which is what produces the 92.38% result. Section 3.1.4 explains why Random Forest is nonetheless retained as the classifier over the *fused* feature space: robustness to the extreme dimensionality and heterogeneous scaling that concatenation produces, resistance to overfitting through bagging, and directly inspectable feature importances that make the token-level SHAP analysis tractable. The Decision Tree is now presented explicitly as a transparent lower bound rather than as a deployment candidate.

**Comment 9.** *"Author should provide the comparison of their proposed approach with previous existing methodologies in graphical manner."*

**Response.** Thank you for this suggestion. Figure 10 presents a bar chart comparing accuracy across eight published studies alongside both proposed models, with the existing studies, the 22-class model and the deployed hybrid distinguished by color and each bar labelled with its value. We have also added a short paragraph stating two caveats plainly: the compared studies use different datasets, so the comparison is indicative rather than a controlled head-to-head; and the compared studies address binary classification, whose random baseline differs from a ten-way task by a factor of five. We prefer to state these limits ourselves rather than leave the figure to be read uncritically.

**Reviewer 3**

We thank Reviewer 3 for an exceptionally thorough review and for the generous summary of the work's contribution. The ten comments were the most methodologically demanding of the three reviews, and addressing them has improved the scientific rigor of the paper considerably.

**Comment 1.** *"The novelty of the proposed approach should be articulated more explicitly in comparison with the most recent studies on multi-class AI source attribution."*

**Response.** Thank you. Two changes address this. Section 1.1 states three specific novelty claims in falsifiable form, each linked to the experiment that tests it. Section 2.6 then contrasts the work point by point with the four closest prior studies on four dimensions: generator coverage, where we evaluate 22 sources against two to six in prior work; treatment of hybrid content, where we separate four collaboration workflows against a single mixed category elsewhere; fusion strategy, where we combine lexical and contextual representations rather than selecting between them; and deployment, which none of the cited attribution studies report.

**Comment 2.** *"The manuscript would benefit from a more comprehensive description of the dataset generation process, including prompt selection strategy, topic diversity, quality control, and measures taken to minimize prompt-induced bias."*

**Response.** Thank you for enumerating these so precisely; we have addressed all four in Section 3.1.1. Prompt selection is covered by the four-step authoring and screening protocol. Topic diversity is defined along three explicit axes and tabulated in Table 1. Quality control covers near-duplicate removal by similarity thresholding, discarding of truncated generations and refusal responses, encoding normalization and label re-verification. On prompt-induced bias specifically, we describe two measures and the reasoning behind each: because identical prompts are issued to every source, topical signal is shared across classes and cannot by itself drive attribution; and because prompts were screened to remove vendor-specific phrasing, no class receives a lexical cue from the prompt. We also describe the prompt-aware splitting strategy that prevents prompt leakage between partitions. Since these procedures are best assessed against the material itself, we note that the corpus has now been publicly released as the TXD-22 dataset at https://doi.org/10.17632/prcjcggtjf.1, so that the curation decisions described here can be verified directly rather than taken on trust.

**Comment 3.** *"Additional statistical analyses such as confidence intervals, significance testing, or repeated cross-validation would strengthen the credibility of the reported performance."*

**Response.** Thank you; all three have been added. Repeated cross-validation is now the primary protocol, with prompt-aware ten-fold cross-validation documented in Section 3.1.7, so every reported figure is an average over ten disjoint evaluations. Table 9 reports 95% confidence intervals for seven configurations, computed by the normal approximation to the binomial with the formula and test sizes stated. Section 4.5 shows that the proposed model's interval, [91.86, 92.90], does not overlap that of any baseline, the nearest being DeBERTa at [73.80, 75.50]. We also note in the same section that the intervals for DeBERTa and RoBERTa overlap substantially, so their 0.28-point difference should not be read as a meaningful ranking, a distinction that point estimates alone would have concealed, and which we state explicitly to avoid over-claiming.

**Comment 4.** *"The experimental evaluation should include stronger benchmarking against recent transformer-based fine-tuned attribution models rather than relying primarily on feature extraction approaches."*

**Response.** Thank you for this; the reliance on frozen feature extraction was a genuine methodological weakness. Table 6 now reports five fine-tuned transformer models (DistilBERT, BERT, ALBERT, DeBERTa and RoBERTa) trained on identical data with identical splits, establishing DeBERTa at 74.65% as the benchmark the proposed method must exceed. Section 4.1.2 also uses these results to test the conjecture offered in the original manuscript, confirming that the weak transformer performance in Table 3 was a consequence of the frozen-extractor protocol rather than of the architectures themselves.

**Comment 5.** *"A detailed confusion matrix and per-class precision, recall, and F1-scores should be presented to reveal which language models are frequently confused."*

**Response.** Thank you for this request, which produced one of the more interesting findings in the revision. Table 8 gives per-class precision, recall, F1, AUC and support for all ten classes, together with macro averages; Figure 5 presents the confusion matrix and Figure 6 the per-class ROC curves. Section 4.4 analyses the pattern rather than merely displaying it. The classes with lowest separability are those involving human editing of AI drafts (AUC 0.80 and 0.83), while those involving an AI refinement pass reach 0.99. This asymmetry is interpretable: AI refinement imposes a consistent, highly detectable signature, whereas human editing is idiosyncratic and partial, degrading the AI signature without imposing a consistent replacement. We also note its practical consequence for users, namely that some SiBot verdicts are considerably more reliable than others, which is why the interface returns the full probability distribution rather than a single label.

**Comment 6.** *"The discussion of generalization remains limited, and the authors should evaluate the model using unseen prompts, cross-domain datasets, or newly released language models."*

**Response.** Thank you. Section 4.7 now addresses generalisation at three levels, and we have tried to be exact about which are demonstrated and which are not. On unseen prompts, every reported figure is already an unseen-prompt result, because prompt-aware partitioning ensures no prompt appears in both training and test folds; had prompt-specific content been driving classification, performance would have collapsed under this constraint. On topical and task variation, the corpus spans six domains and five task types, which establishes robustness across the covered range but not transfer to domains outside it, and we name the absent domains rather than imply coverage. On unseen generators and temporal drift, the honest position is that this remains open: we have not evaluated against models released after corpus construction, and given the RAID findings we do not assume our accuracy holds for them. This is recorded as the principal limitation in Section 5.7 and as a specific future-work commitment in Section 6.

**Comment 7.** *"The manuscript would benefit from a more critical discussion of potential biases, ethical considerations, and possible misuse of AI attribution systems."*

**Response.** Thank you for this comment, which we think has improved the paper as much as any of the technical ones. A new Section 5.6 addresses four areas. On bias, we acknowledge that the corpus is monolingual and institutionally narrow, that prior work has established systematic false positives against non-native English writers, and that we have not evaluated performance disaggregated by author background, so we make no fairness claim. On reliability and appropriate use, we state that at 92.38% accuracy roughly one verdict in thirteen is wrong, that this rate is unacceptable as the basis for a disciplinary finding, and that SiBot's output should prompt inquiry rather than substitute for it. On misuse, we identify four specific pathways, including commercial or political sensitivity of knowing which model an organization uses, disproportionate workplace surveillance, adversarial iteration against a public oracle, and the risk of penalizing legitimate assistive use by writers with disabilities or non-native speakers. On temporal validity, we note that fingerprints have a shelf life and that periodic retraining is a maintenance requirement rather than an enhancement.

**Comment 8.** *"The comparison with commercial tools such as ZeroGPT and QuillBot is informative but lacks methodological rigor because only a limited qualitative comparison is presented; a broader quantitative evaluation… would substantially strengthen the claims."*

**Response.** Thank you; we accept that a single screenshot comparison did not meet the standard the claim required. It has been replaced with a controlled benchmark. Three versions of a response to one generating question were prepared with provenance known by construction, namely purely AI-generated by Gemini, purely human-written, and hybrid human-written then AI-refined, and submitted to five systems: QuillBot, Turnitin, ZeroGPT, TextGuard and SiBot. Confidence scores were recorded across four analysis dimensions for each. Tables 12 to 15 report the material and the results, with an explicit marker for dimensions a tool does not offer at all. The hybrid case is the most revealing: three tools overlooked the AI involvement entirely while a fourth labelled the same text 76% AI, and SiBot alone identified the mixed provenance correctly. We also state plainly that this benchmark, while controlled, remains limited to three texts on one topic, and identify evaluation on a standardised public benchmark such as RAID or OpenTuringBench as necessary further work.

**Comment 9.** *"Greater emphasis should be placed on computational efficiency, training time, memory consumption, and scalability compared with competing methods."*

**Response.** Thank you, particularly given that production deployment is central to our contribution and the original manuscript treated efficiency only qualitatively. Table 10 and Figure 9 now report accuracy against serialized artefact size, inference latency, training time and hardware requirement across six configurations, and Section 4.8 draws the conclusions, including the withdrawal of the unsupported competitiveness claim discussed under Reviewer 1's eighth comment. Section 4.10 covers system scalability, and Section 5.5 adds the deployment constraints specific to serving a two-branch model, notably that the vectorizer, tokenizer configuration and encoder checkpoint must be versioned as a single artefact because silent branch mismatch would yield plausible but meaningless probability distributions.

**Comment 10.** *"The manuscript would benefit from careful language editing to improve readability, reduce repetitive descriptions of the system architecture, and ensure consistency in terminology throughout the paper."*

**Response.** Thank you for this. The manuscript has been edited throughout. The architecture description has been condensed and de-duplicated across Sections 3.2.1 to 3.2.5, where the original repeated the technology stack in three separate places. Terminology has been standardized: we now use "provenance class", "attribution" and "hybrid workflow" consistently, where the original alternated between several formulations. We have also corrected the internally inconsistent class-count statements in the original manuscript, which variously described the corpus as containing 22 AI sources plus a hybrid class and as containing 22 classes in total; the revised text states consistently that there are 21 AI sources plus one hybrid class, totaling 22.
