# Supplementary material for: SiBot: A hybrid framework for fine-grained semantic attribution of AI-generated text and its production deployment
Source: PLoS One. 2026 Sep 8;21(9):e0357405. doi: 10.1371/journal.pone.0357405 (PMC13552777; doi:10.1371/journal.pone.0357405)
Supplement: Peer review history

# Peer review: Decision Letter 0

**DOI:** 10.1371/journal.pone.0357405.r001

**Authors:** Toqir Rana

15 Jul 2026

Dear Dr. Iqbal,

Thank you for submitting your manuscript to PLOS ONE. After careful consideration, we feel that it has merit but does not fully meet PLOS ONE’s publication criteria as it currently stands. Therefore, we invite you to submit a revised version of the manuscript that addresses the points raised during the review process.

Please submit your revised manuscript by Aug 29 2026 11:59PM. If you will need more time than this to complete your revisions, please reply to this message or contact the journal office at plosone@plos.org. When you're ready to submit your revision, log on to https://www.editorialmanager.com/pone/ and select the 'Submissions Needing Revision' folder to locate your manuscript file.

- A letter that responds to each point raised by the academic editor and reviewer(s). You should upload this letter as a separate file labeled 'Response to Reviewers'.
- A marked-up copy of your manuscript that highlights changes made to the original version. You should upload this as a separate file labeled 'Revised Manuscript with Track Changes'.
- An unmarked version of your revised paper without tracked changes. You should upload this as a separate file labeled 'Manuscript'.

If you would like to make changes to your financial disclosure, please include your updated statement in your cover letter. Guidelines for resubmitting your figure files are available below the reviewer comments at the end of this letter.

If applicable, we recommend that you deposit your laboratory protocols in protocols.io to enhance the reproducibility of your results. Protocols.io assigns your protocol its own identifier (DOI) so that it can be cited independently in the future. For instructions see: https://journals.plos.org/plosone/s/submission-guidelines#loc-laboratory-protocols. Additionally, PLOS ONE offers an option for publishing peer-reviewed Lab Protocol articles, which describe protocols hosted on protocols.io. Read more information on sharing protocols at https://plos.org/protocols?utm_medium=editorial-email&utm_source=authorletters&utm_campaign=protocols.

As the corresponding author, your ORCID iD is verified in the submission system and will appear in the published article. PLOS supports the use of ORCID, and we encourage all coauthors to register for an ORCID iD and use it as well. Please encourage your coauthors to verify their ORCID iD within the submission system before final acceptance, as unverified ORCID iDs will not appear in the published article. *Only*  the individual author can complete the verification step; PLOS staff *cannot*  verify ORCID iDs on behalf of authors.

We look forward to receiving your revised manuscript.

Kind regards,

Toqir Rana, Ph.D.

Academic Editor

PLOS One

Journal Requirements:

When submitting your revision, we need you to address these additional requirements.

1. Please ensure that your manuscript meets PLOS ONE's style requirements, including those for file naming. The PLOS ONE style templates can be found at

https://journals.plos.org/plosone/s/file?id=wjVg/PLOSOne_formatting_sample_main_body.pdf and

https://journals.plos.org/plosone/s/file?id=ba62/PLOSOne_formatting_sample_title_authors_affiliations.pdf

2. Please note that PLOS One has specific guidelines on code sharing for submissions in which author-generated code underpins the findings in the manuscript. In these cases, we expect all author-generated code to be made available without restrictions upon publication of the work. Please review our guidelines at https://journals.plos.org/plosone/s/materials-and-software-sharing#loc-sharing-code and ensure that your code is shared in a way that follows best practice and facilitates reproducibility and reuse.

3.PLOS requires an ORCID iD for the corresponding author in Editorial Manager on papers submitted after December 6th, 2016. Please ensure that you have an ORCID iD and that it is validated in Editorial Manager. To do this, go to ‘Update my Information’ (in the upper left-hand corner of the main menu), and click on the Fetch/Validate link next to the ORCID field. This will take you to the ORCID site and allow you to create a new iD or authenticate a pre-existing iD in Editorial Manager.

4. We note that you have indicated that there are restrictions to data sharing for this study. PLOS only allows data to be available upon request if there are legal or ethical restrictions on sharing data publicly. For more information on unacceptable data access restrictions, please see http://journals.plos.org/plosone/s/data-availability#loc-unacceptable-data-access-restrictions.

Before we proceed with your manuscript, please address the following prompts:

a) If there are ethical or legal restrictions on sharing a de-identified data set, please explain them in detail (e.g., data contain potentially identifying or sensitive patient information, data are owned by a third-party organization, etc.) and who has imposed them (e.g., a Research Ethics Committee or Institutional Review Board, etc.). Please also provide contact information for a data access committee, ethics committee, or other institutional body to which data requests may be sent.

b) If there are no restrictions, please upload the minimal anonymized data set necessary to replicate your study findings to a stable, public repository and provide us with the relevant URLs, DOIs, or accession numbers. For a list of recommended repositories, please see

https://journals.plos.org/plosone/s/recommended-repositories. You also have the option of uploading the data as Supporting Information files, but we would recommend depositing data directly to a data repository if possible.

We will update your Data Availability statement on your behalf to reflect the information you provide.

5. If the reviewer comments include a recommendation to cite specific previously published works, please review and evaluate these publications to determine whether they are relevant and should be cited. There is no requirement to cite these works unless the editor has indicated otherwise.

Reviewers' comments:

Reviewer's Responses to Questions

**Comments to the Author**

1. Is the manuscript technically sound, and do the data support the conclusions?

Reviewer #1: Partly

Reviewer #2: Yes

Reviewer #3: Yes

**********

2. Has the statistical analysis been performed appropriately and rigorously?

Reviewer #1: No

Reviewer #2: Yes

Reviewer #3: Yes

**********

3. Have the authors made all data underlying the findings in their manuscript fully available?

The PLOS Data policy

Reviewer #1: No

Reviewer #2: Yes

Reviewer #3: Yes

**********

4. Is the manuscript presented in an intelligible fashion and written in standard English?

Reviewer #1: Yes

Reviewer #2: Yes

Reviewer #3: Yes

**********

Reviewer #1: The manuscript is generally well written, easy to follow, and maintains an appropriate technical tone. However, several important issues should be addressed before the paper can be considered for publication

1. The experimental section is currently limited, with only a single results table presented. Additional experiments and analyses are needed to adequately demonstrate the effectiveness, robustness, and generalizability of the proposed approach

2. The paper lacks sufficient details regarding the data generation and curation process. For example, how prompts were designed to generate or curate the dataset. The authors should provide examples of prompts and describe the methodology used in prompt engineering

3. The paper states that 6,600 samples were used; however, no information is provided regarding data availability. If the dataset cannot be publicly released, representative examples should be included in the manuscript to help readers understand its structure and characteristics

4. The quality and resolution of Figures 1 and 2 should be improved, as some details are difficult to read in their current form. and If created using AI, please disclose that in the acknowledgments for AI usage.

5. The literature review should be strengthened by incorporating recent studies published in 2024, 2025, and 2026. This would help better position the proposed work within the current state of the art

6. Hugging Face repositories, or FastAPI web links should be provided as footnotes

7. An ablation study is recommended to quantify the contribution of different components (configurations) of the proposed framework and demonstrate which design choices are responsible for the observed performance improvements. Moreover, the proposed method should be compared against other multiclass detection/classification approaches. Such comparisons would provide a clearer assessment of its relative strengths and weaknesses.

8. The manuscript claims that lightweight feature-based approaches remain competitive with deep learning methods for production deployment. This claim should be supported with experimental evidence. Currently, authors just discuss about the efficiency of used libraries for front and back end. I suggest, authors should specifically demonstrate that reductions in computational complexity, model size, or deployment cost do not lead to significant degradation in predictive performance.

9. The paper mentions the use of "diverse" data; however, this term is not clearly defined. The authors should explicitly describe what diversity refers to in this context (e.g., mentioning those different domains & prompting methods).

Reviewer #2: 1) The abstract is not expressing the novelty of the proposed approach. The whole abstract is not impressive and needs to be rewritten. Should focus on what is problem, why it is important to be solved. How it is solved and what are findings.

2) Introduction section missing the novelty of the proposed approach; hence, the author is suggested to revise the introduction section.

3) There should be some lines of text between the main and sub heading. This rule should be followed in whole paper.

4) Figure 2 and 3 needs further illustration whereas the tables need consistent font. This rule should be followed in whole paper.

5) Results section needs revision which demonstrates the training as well the testing results. In the meantime, the impacts of each consider parameter in a detailed manner which improves the validity of the approach and quality of the manuscript. Author is requested to add an ablation study which makes the manuscript more impressive.

6) Reference section should comprise of recent years’ contributions.

7) Author should explain the significance of utilizing TF-IDF in feature extraction because more significant techniques are available now which can improve the performance of their proposed approach.

8) provide the logical evidence of utilizing Random Forest and Decision Tree for classification purpose and neglecting the deep learning algorithms. Consideration of the deep learning models may improve the performance of their proposed approach

9) Author should provide the comparison of their proposed approach with previous existing methodologies in graphical manner.

Reviewer #3: The manuscript presents SiBot, a production-ready framework for fine-grained attribution of AI-generated text across 22 language models by integrating TF-IDF feature extraction with Random Forest classification and a publicly accessible web platform. The study addresses an important and timely challenge in AI text forensics by moving beyond conventional binary AI detection toward identifying the likely source model of generated content. The proposed dataset, large-scale experimental evaluation, and end-to-end deployment demonstrate considerable practical effort and provide a useful contribution to the emerging field of AI-generated text attribution. The manuscript is generally well organized and technically sound, with a clear description of the system architecture and implementation. However, several aspects require further clarification and strengthening before publication.

1. The novelty of the proposed approach should be articulated more explicitly in comparison with the most recent studies on multi-class AI source attribution, as the current discussion does not sufficiently distinguish the methodological contribution from existing work.

2. The manuscript would benefit from a more comprehensive description of the dataset generation process, including prompt selection strategy, topic diversity, quality control, and measures taken to minimize prompt-induced bias across language models.

3. Although the reported accuracy is encouraging for a 22-class problem, additional statistical analyses such as confidence intervals, significance testing, or repeated cross-validation would strengthen the credibility of the reported performance.

4. The experimental evaluation should include stronger benchmarking against recent transformer-based fine-tuned attribution models rather than relying primarily on feature extraction approaches, allowing readers to better assess the competitiveness of the proposed method.

5. A detailed confusion matrix and per-class precision, recall, and F1-scores should be presented to reveal which language models are frequently confused and to better understand the limitations of the classifier.

6. The discussion of generalization remains limited, and the authors should evaluate the model using unseen prompts, cross-domain datasets, or newly released language models to demonstrate robustness beyond the constructed dataset.

7. The manuscript would benefit from a more critical discussion of potential biases, ethical considerations, and possible misuse of AI attribution systems, particularly considering the evolving nature of large language models and their frequent updates.

8. The comparison with commercial tools such as ZeroGPT and QuillBot is informative but lacks methodological rigor because only a limited qualitative comparison is presented; a broader quantitative evaluation using standardized benchmark datasets would substantially strengthen the claims.

9. Greater emphasis should be placed on computational efficiency, training time, memory consumption, and scalability compared with competing methods, particularly since production deployment is one of the central contributions of the work.

10. The manuscript would benefit from careful language editing to improve readability, reduce repetitive descriptions of the system architecture, and ensure consistency in terminology throughout the paper.

Overall, the work addresses a highly relevant research problem and demonstrates practical significance, but addressing the above comments would considerably improve its scientific rigor, reproducibility, and impact, making it more suitable for publication.

**********

what does this mean?). If published, this will include your full peer review and any attached files.

If you choose “no”, your identity will remain anonymous but your review may still be made public.

**Do you want your identity to be public for this peer review?**  For information about this choice, including consent withdrawal, please see our Privacy Policy

Reviewer #1: No

Reviewer #2: No

Reviewer #3: No

**********

[NOTE: If reviewer comments were submitted as an attachment file, they will be attached to this email and accessible via the submission site. Please log into your account, locate the manuscript record, and check for the action link "View Attachments". If this link does not appear, there are no attachment files.]

To ensure your figures meet our technical requirements, please review our figure guidelines: https://journals.plos.org/plosone/s/figures

You may also use PLOS’s free figure tool, NAAS, to help you prepare publication quality figures: https://journals.plos.org/plosone/s/figures#loc-tools-for-figure-preparation.

NAAS will assess whether your figures meet our technical requirements by comparing each figure against our figure specifications.

Attachment Submitted filename: PloS One review report.docx

# Author comment: Author response to Decision Letter 1

**DOI:** 10.1371/journal.pone.0357405.r002

29 Jul 2026

Response to Reviewers

Overall Response: We are grateful to the Editor and to all three reviewers for the time and care they have devoted to this manuscript. The comments were detailed, constructive and, in several instances, identified weaknesses that we had not adequately recognized ourselves. Every comment has resulted in a substantive change to the paper. All modified and newly added text is highlighted in yellow in the revised manuscript so that the changes can be located quickly.

Three changes affect the manuscript globally, and we describe them once here rather than repeating them under each comment. First, the deployed SiBot system now serves the 10-class hybrid model (TF-IDF + DeBERTa + Random Forest, 92.38% accuracy) rather than the 22-class model. Both sets of results are reported in full, the reasoning for the deployment choice is set out in Section 3.1.8, and extending deployment to all 22 classes is stated as the leading item of future work. Second, a claim made in the original manuscript, that lightweight feature-based approaches "remain competitive" with deep learning, proved not to be supported by our own data once we ran the additional experiments the reviewers requested. We have withdrawn the claim rather than defend it, and replaced it in Section 4.8 with the narrower statements the evidence does support. Third, the dataset underlying the study has been publicly released as the TXD-22 benchmark at https://doi.org/10.17632/prcjcggtjf.1, and the model controller source code has been made openly available, so that the results reported here are reproducible in full. Fourth, the manuscript has been thoroughly edited for language, repetition and terminological consistency throughout.

Reviewer 1

We thank Reviewer 1 for the encouraging assessment of the writing and technical tone, and for a set of comments that were unusually specific and therefore unusually actionable. The manuscript is considerably stronger for them.

Comment 1. "The experimental section is currently limited, with only a single results table presented. Additional experiments and analyses are needed to adequately demonstrate the effectiveness, robustness, and generalizability of the proposed approach."

Response. Thank you for this comment; it was the single most consequential criticism in the reviews and it prompted a substantial expansion of the work. The experimental section has grown from one table to sixteen tables and fifteen figures. Specifically, we have added Table 4 (five classical machine-learning models), Table 5 (five deep-learning architectures), Table 6 (five fine-tuned transformer models), Table 7 with Figure 3 (a component-wise ablation), Table 8 with Figures 4 to 7 (per-class metrics, confusion matrix and ROC analysis), Table 9 (confidence intervals), Table 10 with Figure 9 (computational efficiency), Table 11 with Figure 10 (comparison against eight published studies), and Tables 12 to 15 (a controlled quantitative benchmark against four commercial detectors). Robustness is addressed through prompt-aware ten-fold cross-validation and a dedicated generalization analysis in Section 4.7. These additions appear in Sections 4.2 to 4.9.

Comment 2. "The paper lacks sufficient details regarding the data generation and curation process… The authors should provide examples of prompts and describe the methodology used in prompt engineering."

Response. Thank you for raising this; the original description was indeed too thin to allow the corpus construction to be assessed or reproduced. Section 3.1.1 now documents the full protocol. The prompt bank comprises 3,000 unique prompts spanning thirty academic domains, each with eight to ten sub-domains, crossed with five task types, and the same 3,000 prompts were put verbatim to every source. The authoring procedure is described in four steps: drafting seed prompts per cell, screening for near-duplication using cosine similarity over TF-IDF vectors with a rejection threshold of 0.85, manual review to remove ambiguity and any vendor-specific or product-specific wording that might bias a particular model family, and finally issuing the frozen prompt verbatim to every source. We also explain why identical prompting matters for the validity of the attribution claim. Table 1 now gives eight verbatim example prompts drawn from across the design.

Comment 3. "The paper states that 6,600 samples were used; however, no information is provided regarding data availability. If the dataset cannot be publicly released, representative examples should be included in the manuscript."

Response. Thank you for this comment, which prompted us to go further than the alternative you offered. Rather than substituting representative examples for release, we have released the dataset itself. The full corpus has been deposited in the Mendeley Data repository under the name TXD-22 and is openly available at https://doi.org/10.17632/prcjcggtjf.1. It is cited in the manuscript as: Iqbal, M. S., & Kashem, M. A. (2026). TXD-22: A large-scale benchmark dataset for multi-class AI-generated and mixed-source text identification (Version 1) [Data set]. Mendeley Data. https://doi.org/10.17632/prcjcggtjf.1. The citation appears as reference [81], as a footnote in Section 3.1.1, in the Data Availability Statement, and in the contributions list in Section 1.1, since public release is itself a contribution of the work. Every result reported in Section 4 is therefore now directly reproducible. We have nonetheless retained the representative examples you suggested: Table 2 presents one sample per class for all ten classes, showing prompt, response and label, with each example deliberately drawn from a different subject domain so that the topical breadth of the corpus is visible at a glance, and Table 12 reproduces the complete benchmark texts verbatim, so that a reader can assess the structure of the corpus without downloading it. We should note that the sample count in the original manuscript was misstated; the corpora comprise 66,000 and 30,000 samples respectively, and this has been corrected throughout.

Comment 4. "The quality and resolution of Figures 1 and 2 should be improved… and if created using AI, please disclose that in the acknowledgments for AI usage."

Response. Thank you for both points. Figures 1 and 2 (the pipeline and architecture diagrams) have been completely redrawn at 600 dpi with substantially enlarged typography, and the architecture diagram now additionally carries a numbered request lifecycle so that the data flow can be followed without reference to the body text. On the disclosure point, we have added a dedicated section, "Acknowledgements and Disclosure of AI Usage", stating the provenance of every figure individually: which were produced with AI-assisted drawing tools and subsequently verified by the authors, which were generated programmatically from the experimental results, which are direct outputs of our own experimental code, and which are unmodified screenshots. The statement also discloses that generative AI systems were used to produce the research corpus itself, which is the subject matter of the study, and for language editing, and confirms that all technical content and conclusions are the authors' own.

Comment 5. "The literature review should be strengthened by incorporating recent studies published in 2024, 2025, and 2026."

Response. Thank you for this; the review was indeed weighted toward work published before 2024. We have added a new Section 2.5 devoted to recent advances, organised around the three developments most relevant to our design decisions. The first is robustness benchmarking, covering RAID (ACL 2024) and MGTBench (CCS 2024), which motivated our prompt-aware splitting protocol and the candid generalisation analysis in Section 4.7. The second is efficient zero-shot detection, covering Fast-DetectGPT (ICLR 2024) and Binoculars (ICML 2024), which we discuss as strong binary detectors that are structurally unable to perform attribution. The third is the consolidation of attribution as a distinct task, covering the SIGKDD Explorations survey (2025), OpenTuringBench (EMNLP 2025), LLM code stylometry (2025), LLM-as-a-Coauthor (2024), EditLens (2025) and recent linguistic-feature work. Fifteen references from 2024 to 2026 have been added and integrated into the argument rather than merely appended.

Comment 6. "Hugging Face repositories, or FastAPI web links should be provided as footnotes."

Response. Thank you for this suggestion, which has made the paper considerably more useful to anyone wishing to build on it. Four footnotes have been added. The first, in the Introduction, gives the live application at https://www.sibot.us/. The second gives the Hugging Face Spaces inference service together with the model controller repository. The third, in Section 3.2.3, gives the interactive FastAPI/OpenAPI documentation served at the /docs path of the hosted service, where every endpoint described in that section can be exercised directly. The fourth, in Section 3.1.1, gives the dataset. The two code and data resources are cited in full as follows. The source repository: Iqbal, M. S., & Kashem, M. A. (2026). SiBot model controller [Computer software]. GitHub. Retrieved July 27, 2026, from https://github.com/XDR-SAM/SIBOT_Model_Controller.git, listed as reference [82], which contains the model controller source code, the deployment configuration and the supplementary material. The dataset: Iqbal, M. S., & Kashem, M. A. (2026). TXD-22: A large-scale benchmark dataset for multi-class AI-generated and mixed-source text identification (Version 1) [Data set]. Mendeley Data. https://doi.org/10.17632/prcjcggtjf.1, listed as reference [81]. Both are also given in the Data Availability Statement so that they are visible to readers who consult that section rather than the footnotes.

Comment 7. "An ablation study is recommended… Moreover, the proposed method should be compared against other multiclass detection/classification approaches."

Response. Thank you for this recommendation, which materially improved the evidential basis of the paper. Table 7 and Figure 3 now present a component-wise ablation on identical data and splits, comparing the proposed configuration against its lexical branch alone (58.08%), its contextual branch alone (74.65%), and two alternative fusion designs (67.82% and 68.56%). The proposed configuration reaches 92.38%. We draw particular attention to the fact that both alternative fusion designs fall below the standalone transformer baseline, which demonstrates that fusion is not automatically beneficial and that the specific combination of branches and classifier is what carries the result. On the second part of the comment, Table 11 and Figure 10 compare the method against eight published detection and classification studies, with the commensurability caveats stated explicitly in the accompanying text.

Comment 8. "The manuscript claims that lightweight feature-based approaches remain competitive with deep learning methods for production deployment. This claim should be supported with experimental evidence… authors should specifically demonstrate that reductions in computational complexity, model size, or deployment cost do not lead to significant degradation in predictive performance."

Response. Thank you for pressing on this. We tested the claim as you asked, and we must report that it did not survive the test: on the 10-class task, TF-IDF with Random Forest attains 58.08% against 74.65% for a fine-tuned transformer, which is not competitive by any reasonable reading. We have therefore withdrawn the claim from the abstract, the introduction and the conclusion rather than attempt to defend it. In its place, Section 4.8 and Table 10 report accuracy against artefact size, inference latency, training time and hardware requirement, and state the two narrower claims that the evidence does support: that on the 22-class task sparse lexical features outperform frozen transformer embeddings at roughly a third of the artefact size, which is a genuine result about frozen-extractor pipelines specifically; and that the deployed hybrid attains the highest accuracy of any configuration evaluated while remaining servable on CPU-only infrastructure at roughly half the footprint of a fine-tuned DeBERTa. The efficiency argument thus survives, but as a property of the hybrid rather than of the lightweight branch alone. Figure 9 plots this trade-off. We are grateful for a comment that led us to correct a genuine overstatement.

Comment 9. "The paper mentions the use of 'diverse' data; however, this term is not clearly defined."

Response. Thank you; the term was indeed used loosely. Section 3.1.1 now defines diversity along three explicit axes. Topical diversity means coverage of thirty academic domains, each broken down into eight to ten sub-domains, spanning engineering, the natural sciences, the social sciences, law, medicine, the humanities and the creative and media disciplines. The full distribution is now given as Table 1. Task diversity means coverage of five task types within each domain: factual explanation, comparative analysis, procedural description, opinion or argumentative writing, and summarization. Structural diversity means deliberate variation in prompt length and specificity, from single-clause questions of under ten words to multi-constraint instructions exceeding fifty words. Table 1 sets out the full domain and sub-domain distribution, and Table 2 illustrates the same breadth by drawing its ten class examples from ten different domains of that table rather than from a single field. We have also made explicit that the same 3,000 prompts were put verbatim to every generative source, so that the classes differ only in what produced the response and not in what was asked.

Reviewer 2

We thank Reviewer 2 for a set of comments that addressed both the substance of the work and its presentation. The comments on the abstract, on the justification of our methodological choices, and on the treatment of deep learning were particularly valuable, and each has led to significant revision.

Comment 1. "The abstract is not expressing the novelty of the proposed approach. The whole abstract is not impressive and needs to be rewritten. Should focus on what is problem, why it is important to be solved. How it is solved and what are findings."

Response. Thank you for this direct assessment, which we accept. The abstract has been rewritten from scratch and now follows precisely the structure you specify. It opens by stating the problem, namely that deployed detectors answer only a binary question. It then establishes why this matters, namely that forensic and academic-integrity settings require knowing which model produced a text and whether a human was involved, neither of which a single probability can express. It describes the solution: two purpose-built corpora constructed from a controlled prompt bank, and a hybrid architecture fusing sparse lexical and contextual representations. And it closes with quantified findings, including the 92.38% result, the 17.73-point margin over the strongest transformer baseline, the ablation outcome, and the comparison against four commercial detectors.

Comment 2. "Introduction section missing the novelty of the proposed approach; hence, the author is suggested to revise the introduction section."

Response. Thank you. The introduction has been revised in two respects. A new paragraph states the specific problem precisely and identifies the three obstacles that have prevented the reformulation from being realized. More importantly, a new Section 1.1, "Novelty and Contributions", states three specific and testable novelty claims: that lexical and contextual evidence are complementary rather than redundant; that human-AI collaboration is separable into distinguishable workflow classes rather than a single hybrid bucket; and that attribution-grade granularity is achievable within production constraints. Each claim is cross-referenced to the experiment that tests it, so the novelty is stated as something falsifiable rather than asserted.

Comment 3. "There should be some lines of text between the main and sub heading. This rule should be followed in whole paper."

Response. Thank you for

Attachment Submitted filename: Reviewer Response.docx

# Peer review: Decision Letter 1

**DOI:** 10.1371/journal.pone.0357405.r003

**Authors:** Toqir Rana

17 Aug 2026

SiBot: A Hybrid Framework for Fine-Grained Semantic Attribution of AI-Generated Text and Its Production Deployment

PONE-D-26-15376R1

Dear Dr. Iqbal,

We’re pleased to inform you that your manuscript has been judged scientifically suitable for publication and will be formally accepted for publication once it meets all outstanding technical requirements.

Within one week, you’ll receive an e-mail detailing the required amendments. When these have been addressed, you’ll receive a formal acceptance letter and your manuscript will be scheduled for publication.

An invoice will be generated when your article is formally accepted. Please note, if your institution has a publishing partnership with PLOS and your article meets the relevant criteria, all or part of your publication costs will be covered. Please make sure your user information is up-to-date by logging into Editorial Manager at Editorial Manager® and clicking the ‘Update My Information' link at the top of the page. For questions related to billing, please contact billing support.

If your institution or institutions have a press office, please notify them about your upcoming paper to help maximize its impact. If they’ll be preparing press materials, please inform our press team as soon as possible -- no later than 48 hours after receiving the formal acceptance. Your manuscript will remain under strict press embargo until 2 pm Eastern Time on the date of publication. For more information, please contact onepress@plos.org.

Kind regards,

Toqir Rana

Academic Editor

PLOS One

Additional Editor Comments (optional):

Reviewers' comments:

Reviewer's Responses to Questions

**Comments to the Author**

Reviewer #2: All comments have been addressed

Reviewer #3: All comments have been addressed

**********

2. Is the manuscript technically sound, and do the data support the conclusions?

Reviewer #2: Yes

Reviewer #3: Yes

**********

3. Has the statistical analysis been performed appropriately and rigorously?

Reviewer #2: Yes

Reviewer #3: Yes

**********

4. Have the authors made all data underlying the findings in their manuscript fully available?

The PLOS Data policy

Reviewer #2: Yes

Reviewer #3: Yes

**********

5. Is the manuscript presented in an intelligible fashion and written in standard English?

Reviewer #2: Yes

Reviewer #3: Yes

**********

Reviewer #2: (No Response)

Reviewer #3: The authors have addressed all the comments/comments satisfactorily. Hence the article may be accepted for publication.

**********

what does this mean?). If published, this will include your full peer review and any attached files.

If you choose “no”, your identity will remain anonymous but your review may still be made public.

**Do you want your identity to be public for this peer review?**  For information about this choice, including consent withdrawal, please see our Privacy Policy

Reviewer #2: No

Reviewer #3: No

**********

# Editor report: Acceptance letter

**DOI:** 10.1371/journal.pone.0357405.r004

**Authors:** Toqir Rana

PONE-D-26-15376R1

PLOS One

Dear Dr. Iqbal,

I'm pleased to inform you that your manuscript has been deemed suitable for publication in PLOS One. Congratulations! Your manuscript is now being handed over to our production team.

At this stage, our production department will prepare your paper for publication. This includes ensuring the following:

* All references, tables, and figures are properly cited

* All relevant supporting information is included in the manuscript submission,

* There are no issues that prevent the paper from being properly typeset

You will receive further instructions from the production team, including instructions on how to review your proof when it is ready. Please keep in mind that we are working through a large volume of accepted articles, so please give us a few days to review your paper and let you know the next and final steps.

Lastly, if your institution or institutions have a press office, please let them know about your upcoming paper now to help maximize its impact. If they'll be preparing press materials, please inform our press team within the next 48 hours. Your manuscript will remain under strict press embargo until 2 pm Eastern Time on the date of publication. For more information, please contact onepress@plos.org.

You will receive an invoice from PLOS for your publication fee after your manuscript has reached the completed accept phase. If you receive an email requesting payment before acceptance or for any other service, this may be a phishing scheme. Learn how to identify phishing emails and protect your accounts at https://explore.plos.org/phishing.

If we can help with anything else, please email us at customercare@plos.org.

Thank you for submitting your work to PLOS One and supporting open access.

Kind regards,

PLOS One Editorial Office Staff

on behalf of

Dr. Toqir Rana

Academic Editor

PLOS One
